# Adenylyl Cyclase and Protein Kinase A Play Redundant and Distinct Roles in Growth, Differentiation, Antifungal Drug Resistance, and Pathogenicity of *Candida auris*

**DOI:** 10.1128/mBio.02729-21

**Published:** 2021-10-19

**Authors:** Ji-Seok Kim, Kyung-Tae Lee, Myung Ha Lee, Eunji Cheong, Yong-Sun Bahn

**Affiliations:** a Department of Biotechnology, College of Life Science and Biotechnology, Yonsei University, Seoul, South Korea; University of British Columbia

**Keywords:** *C. auris*, cyclic AMP, cAMP, multidrug resistance, pseudohyphae, biofilm, ploidy change, virulence, human fungal pathogen

## Abstract

Candida auris is a globally emerging multidrug-resistant fungal pathogen. Its pathogenicity-related signaling networks are largely unknown. Here, we characterized the pathobiological functions of the cyclic AMP (cAMP)/protein kinase A (PKA) signaling pathway in C. auris. We focused on adenylyl cyclase (*CYR1*), the PKA regulatory subunit (*BCY1*), and the PKA catalytic subunits (*TPK1* and *TPK2*). We concluded that PKA acts both dependently and independently of Cyr1 in C. auris. Tpk1 and Tpk2 have major and minor roles, respectively, in PKA activity and functions. Both Cyr1 and PKA promote growth, thermotolerance, filamentous growth, and resistance to stress and antifungal drugs by regulating expression of multiple effector genes. In addition, Cyr1 and PKA subunits were involved in disinfectant resistance of C. auris. However, deletion of both *TPK1* and *TPK2* generally resulted in more severe defects than *CYR1* deletion, indicating that Cyr1 and PKA play redundant and distinct roles. Notably, Tpk1 and Tpk2 have redundant but Cyr1-independent roles in haploid-to-diploid cell transition, which increases virulence of C. auris. However, Tpk1 and Tpk2 often play opposing roles in formation of biofilms and the cell wall components chitin and chitosan. Surprisingly, deletion of *CYR1* or *TPK1/TPK2*, which resulted in severe *in vitro* growth defects at 37°C, did not attenuate virulence, and *BCY1* deletion reduced virulence of C. auris in a systemic murine infection model. In conclusion, this study provides comprehensive insights into the role of the cAMP/PKA pathway in drug resistance and pathogenicity of C. auris and suggests a potential therapeutic option for treatment of C. auris*-*mediated candidemia.

## INTRODUCTION

The fungus Candida auris is considered an emerging human pathogen of global concern. It was first isolated in Japan in 2009 (1) and has since been detected in six continents and more than 30 countries (2). Four clades have been identified in the species. Clade I was first identified in Europe and the Middle East, clade II in East Asia (including Korea and Japan), clade III in Africa, and clade IV in America. In addition, a potential fifth clade has been reported in Iran (3, 4). Most members of these clades cause invasive candidiasis, which has been reported in Asia, South Africa, and South America (5). In a health care setting, C. auris can form biofilms on the surface of medical devices or on the surface of patient’s skin. These are problematic since they are difficult to eliminate and, consequently, may result in nosocomial infections (6). The fungus has been isolated in the lungs, blood, livers, kidneys, and ears of infected individuals. Infection is of particular concern as C. auris is resistant to many of the existing antifungal drugs, including azoles (7). The U.S. Centers for Disease Control and Prevention (CDC) recommends treating C. auris infections with echinocandins, but some strains are also resistant to these (8). Because of these problems, the mortality rate from candidemia is exceptionally high, ranging from 30 to 60% (9).

Despite its importance in global health, the virulence mechanism and related signaling pathways remain largely unknown for C. auris. Among known signaling pathways, the evolutionarily conserved cyclic AMP (cAMP) pathway is generally required for the pathogenicity of human and plant fungal pathogens (10–13). In response to various environmental cues, such as glucose or amino acids, adenylyl cyclase is activated through heterotrimeric or monomeric guanine nucleotide-binding proteins (G proteins) and a G-protein-coupled receptor. When activated, adenylyl cyclase converts ATP to cAMP (14). The cAMP produced then binds to the regulatory subunit of protein kinase A (PKA) to release catalytic subunits of PKA, which activate downstream transcription factors and other effectors involved in various biological reactions. In Candida albicans, the cAMP/PKA pathway modulates stress response, vegetative and hyphal growth, virulence, and biofilm formation (15). In addition, cAMP is involved in the expression of the genes *CDR*, *MDR*, *ERG*, and *TAC1*, which are related to resistance of *Candida* species to antifungal drugs (16, 17). Given this background in related species, we were led to examine the pathobiological role of the cAMP/PKA pathway in C. auris, which to our knowledge has not been characterized.

In this study, we identified the genes encoding adenylyl cyclase (*CYR1*), the regulatory subunit of PKA (*BCY1*), and the catalytic subunits of PKA (*TPK1* and *TPK2*) in C. auris and functionally analyzed them through reverse genetics and phenotypic analyses. Here, we found that PKA plays Cyr1-dependent and -independent roles in regulating temperature-dependent growth, the formation of pseudohyphae, stress responses, ploidy switching, and resistance to antifungal drugs and disinfectants in C. auris. Most importantly, we found that hyperactivation, but not inhibition, of the cAMP/PKA pathway significantly reduced the virulence of C. auris.

We believe that our study provides important insights into the virulence-regulating signaling pathway of C. auris. We hope this insight will aid the development of novel, effective treatments for candidemia caused by C. auris infection.

## RESULTS

### Construction of *Candida auris* deletion mutants and complemented strains of genes involved in the cAMP/PKA pathway.

The goal of this study was to functionally characterize the cAMP/PKA pathway in C. auris. We first searched for genes encoding adenylyl cyclase and the catalytic and regulatory subunits of PKA in the *Candida* Genome Database (http://www.candidagenome.org). The four proteins in C. auris that had greatest identity to the corresponding protein in the C. albicans cAMP/PKA pathway were Cyr1 (B9J08_004540), Bcy1 (B9J08_002818), Tpk1 (B9J08_004030), and Tpk2 (B9J08_002788) (see Fig. S1). We concluded that the C. auris PKA complex consists of two catalytic subunits (Tpk1 and Tpk2) and a single regulatory subunit (Bcy1), as does the PKA complex from C. albicans.

10.1128/mBio.02729-21.1 FIG S1 Phylogenetic analyses of fungal adenylyl cyclases and PKA subunits. Phylogenetic trees of fungal orthologs for adenylyl cyclase (A), the PKA regulatory subunit (B), and PKA catalytic subunits (C and D) were retrieved from Candida Genome Database (http://www.candidagenome.org/). Download FIG S1, PDF file, 0.05 MB. Copyright © 2021 Kim et al. 2021 Kim et al. https://creativecommons.org/licenses/by/4.0/ This content is distributed under the terms of the Creative Commons Attribution 4.0 International license.

Next, we attempted to construct gene deletion mutants through homologous recombination. We constructed split-gene disruption cassettes with 5′ and 3′ homologous regions of 0.6 to 1.0 kb and the nourseothricin resistance marker (*CaNAT*) or the hygromycin B resistance marker (*CaHYG*). These were introduced into the wild-type (WT) strain by electroporation (see Fig. S2). We validated the phenotypic traits of the gene deletion mutants by constructing complemented strains in which the WT allele was either ectopically integrated (*cyr1*Δ+*CYR1*) or reintegrated into its native locus (*bcy1*Δ::*BCY1*, *tpk1*Δ::*TPK1*, and *tpk2*Δ::*TPK2*) (see Fig. S3).

10.1128/mBio.02729-21.2 FIG S2 Construction and validation of gene deletion mutants in Candida auris. Target genes were replaced with gene deletion cassette containing the nourseothricin resistance gene (*NAT*^r^) or the hygromycin resistance gene (*HYG*^r^) delivered by electroporation in C. auris (see Materials and Methods). Homologous recombination strategies between the WT gene and the deletion cassette are illustrated graphically (left panels). Transformants were confirmed by diagnostic PCR (center panels) and Southern blot analysis (right panels). All primer sets were listed in Table S2. (A) The *CYR1* gene in WT was replaced with the *NAT*^r^ marker. The genomic DNA of WT and *cyr1*Δ mutants was digested by EcoRV for Southern blot analysis. (B) The *BCY1* gene in WT was replaced with the *NAT*^r^ marker. The genomic DNA of WT and *bcy1*Δ mutants was digested by HincII for Southern blot analysis. (C) The *TPK1* gene in WT was replaced with the *NAT*^r^ marker. The genomic DNA of WT and *tpk1*Δ mutants was digested by XhoI for Southern blot analysis. (D) The *TPK2* gene in WT was replaced with the *NAT*^r^ marker. The genomic DNA of WT and *tpk2*Δ mutants was digested by HincII for Southern blot analysis. (E) The *TPK1* gene in the *tpk2*Δ mutant was replaced with *HYG*^r^ marker. The genomic DNA WT and *tpk1*Δ *tpk2*Δ mutants was digested by HincII for Southern blot analysis. Download FIG S2, PDF file, 0.2 MB. Copyright © 2021 Kim et al. 2021 Kim et al. https://creativecommons.org/licenses/by/4.0/ This content is distributed under the terms of the Creative Commons Attribution 4.0 International license.

10.1128/mBio.02729-21.3 FIG S3 Validation of complemented strains for *cyr1*Δ, *bcy1*Δ, *tpk1*Δ, and *tpk2*Δ mutants. Ectopic integration of a WT gene was confirmed by PCR using primer pairs in exon regions, and targeted integration of a WT gene into its native locus was confirmed by PCR using primers in the exon regions paired with primers outside the homologous region. All primer sets are listed in Table S2. Diagnostic PCR showed the results of ectopic integration of the *CYR1* gene in the *cyr1*Δ mutant (*cyr1*Δ+*CYR1*) (A), targeted integration of the *BCY1* gene in the *bcy1*Δ mutant (*bcy1*Δ::*BCY1*) (B), targeted integration of the *TPK1* gene in the *tpk1*Δ mutant (*tpk1*Δ::*TPK1*) (C), and targeted integration of the *TPK2* gene in the *tpk2*Δ mutant (*tpk2*Δ::*TPK2*) (D). Each expected size is indicated under the corresponding electrophoresis image. Download FIG S3, PDF file, 0.09 MB. Copyright © 2021 Kim et al. 2021 Kim et al. https://creativecommons.org/licenses/by/4.0/ This content is distributed under the terms of the Creative Commons Attribution 4.0 International license.

### The role of the cAMP/PKA signaling pathway in growth of *C. auris*.

The cAMP/PKA signaling pathway is important in growth and thermotolerance of fungal species (18). In particular, C. auris exhibits much greater thermotolerance (can grow well up to 42°C) than other *Candida* species (1). This is of particular concern since warming associated with global climate change could enhance the importance of this emerging pathogen (19).

**FIG 1:**
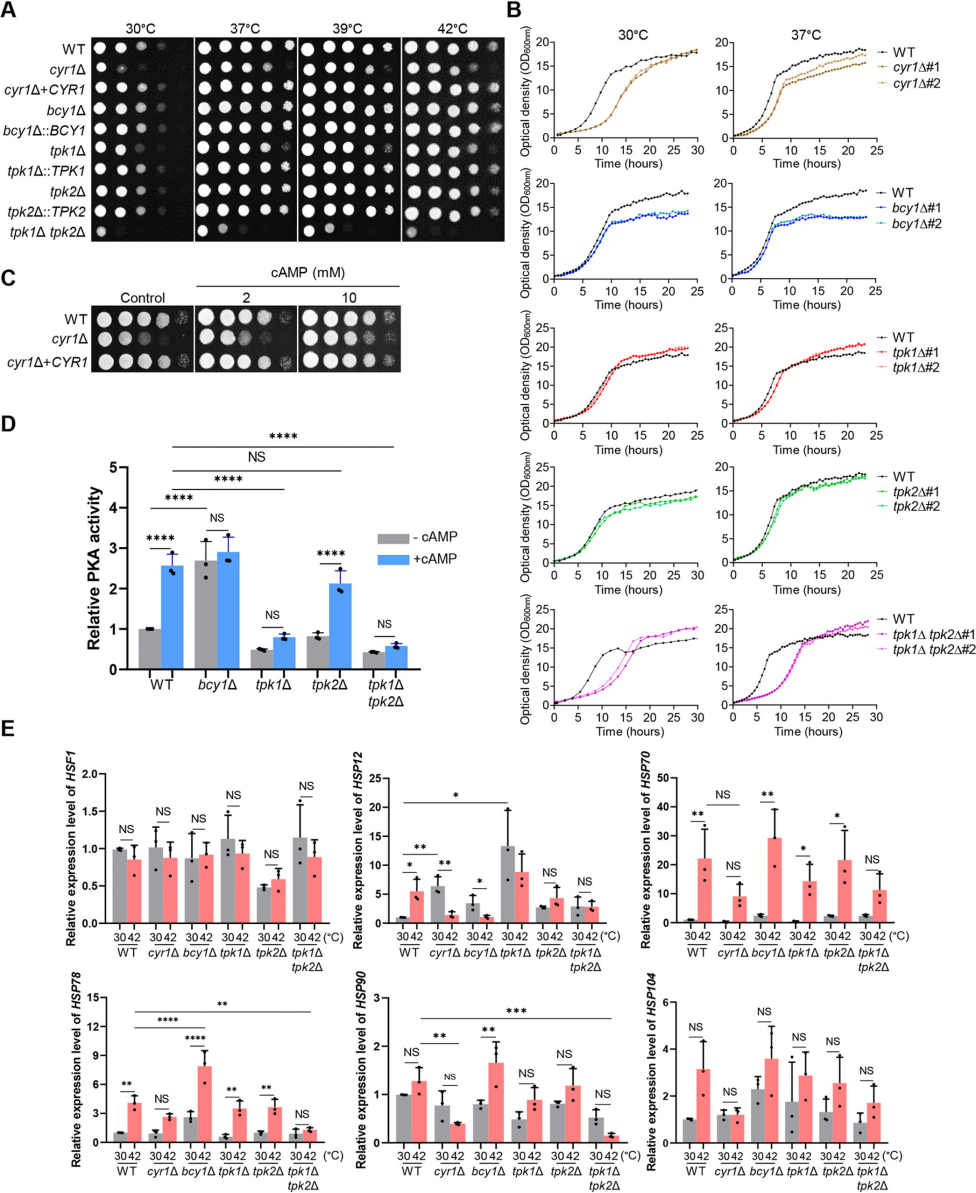
Role of adenylyl cyclase 1 (Cyr1) and protein kinase A (PKA) in the growth and thermotolerance of Candida auris. (A) Qualitative spot assays for measuring the growth and thermotolerance of the WT and the *cyr1*Δ (YSBA21), *cyr1*+*CYR1* (YSBA38), *bcy1*Δ (YSBA4), *bcy1*Δ::*BCY1* (YSBA29), *tpk1*Δ (YSBA13), *tpk1*Δ::*TPK1* (YSBA36), *tpk2*Δ (YSBA17), *tpk2*Δ::*TPK2* (YSBA26), *tpk1*Δ *tpk2*Δ (YSBA24) mutants. Cells were cultured in liquid medium at 30°C overnight, serially diluted (1 to 10^4^), and spotted onto plates. The plates were incubated for 1 day at 30, 37, 39, or 42°C and photographed. A representative image from three independent experiments is shown. (B) Quantitative growth rates of the WT and the *cyr1*Δ#1 (YSBA21), *cyr1*Δ#2 (YSBA23), *bcy1*Δ#1 (YSBA4), *bcy1*Δ#2 (YSBA6), *tpk1*Δ#1 (YSBA13), *tpk1*Δ#2 (YSBA14), *tpk2*Δ#1 (YSBA16), *tpk2*Δ#2 (YSBA17), *tpk1*Δ *tpk2*Δ#1 (YSBA24), *tpk1*Δ *tpk2*Δ#2 (YSBA25) mutants. Growth was monitored at 30 and 37°C by measuring the OD_600_ with a multichannel bioreactor (Biosan Laboratories, Inc., Warren, MI) for 20 to 25 h. Each curve is a single representative of two independent experiments. (C) Growth complementation of the adenylyl cyclase mutant with exogenous cAMP. WT or mutant *cyr1*Δ (YSBA21) and *cyr1*Δ+*CYR1* (YSBA38) strain cells were cultured in liquid medium at 30°C overnight, serially diluted (1 to 10^4^), and spotted onto plates with media supplemented with 2 mM or 10 mM cAMP. The plates were incubated for 1 day at 30°C and photographed. (D) PKA assay. PKA activity was measured by using crude cell extracts from the WT or the *bcy1*Δ (YSBA4), *tpk1*Δ (YSBA13), *tpk2*Δ (YSBA17), and *tpk1*Δ *tpk2*Δ (YSBA24) mutants in the presence or absence of 10 μM cAMP. Three independent biological experiments were performed. (E) qRT-PCR analysis of thermotolerance-related genes in the WT and in *cyr1*Δ (YSBA21), *bcy1*Δ (YSBA4), *tpk1*Δ (YSBA13), *tpk2*Δ (YSBA17), and *tpk1*Δ *tpk2*Δ (YSBA24) mutant strains. Cells were cultured overnight at 30°C in YPD broth and subcultured to an OD_600_ of 0.8 at 30°C in fresh broth extracted for total RNA (referred to as the 30°C sample). A portion was incubated for another 2 h at 42°C, and the total RNA was extracted (the 42°C sample). The expression level of each gene was normalized using *ACT1* as the standard; the fold change was calculated relative to the basal expression level of thermotolerance-related genes in the WT strain. Three independent biological experiments with three technical replicates were performed. (The medium was YPD broth containing 1% yeast extract, 2% peptone, and 2% dextrose; in panels D and E, data points represent the means and error bars represent the standard errors of the mean (SEM). Statistical significance of difference was determined by one-way ANOVA with Bonferroni’s multiple-comparison test (*, *P *<* *0.05; **, *P *<* *0.01; ***, *P *<* *0.001; ****, *P *<* *0.0001; NS, not significant.).

To address the role of the cAMP/PKA pathway in the growth and thermotolerance of C. auris, we compared the growth of *cyr1*Δ, *tpk1*Δ, *tpk2*Δ, *tpk1*Δ *tpk2*Δ, and *bcy1*Δ mutants to that of the WT parental strain B8441 (clade I) and their complemented strains, both qualitatively and quantitatively, at a range of temperatures from 30 to 42°C. It was previously noted (19) that C. auris grew faster at high temperatures (37, 39, and 42°C) than at low temperatures (25 and 30°C). We found that deletion of *CYR1* markedly reduced the growth of C. auris compared to the WT strain, and this was most evident at 30°C. The growth rate was restored to that of the WT by complementation with the WT *CYR1* gene (Fig. 1A). When we measured growth quantitatively, we observed that the *cyr1*Δ mutant did not enter the exponential growth phase as early as the WT strain, although it did have the same cell density as the WT strain after 24 h of incubation (Fig. 1B). It may be that it is necessary for C. auris cells to maintain basal cAMP levels for the cells to enter the exponential growth phase. We tested this idea by adding exogenous cAMP to the media, which partly restored normal growth in the *cyr1*Δ mutant (Fig. 1C).

We next addressed whether the growth defects of the *cyr1*Δ mutant resulted from inactivation of Tpk1 and Tpk2 downstream of Cyr1. Deletion of *TPK1*, but not *TPK2*, reduced growth of C. auris by delaying entry into the exponential growth phase, albeit to a lesser extent than the *cyr1*Δ mutant (Fig. 1A and B). We concluded that Tpk1 is likely a major catalytic subunit of PKA. However, deletion of both *TPK1* and *TPK2* caused more severe growth defects than that of *CYR1* or *TPK1* at all temperatures (37 to 42°C) (Fig. 1A), which may be evidence that Tpk2 has a minor role in growth. Notably, *tpk1*Δ, *tpk2*Δ, and *tpk1*Δ *tpk2*Δ mutants made delayed entry into the exponential growth phase but had higher cell density at the stationary growth phase than the WT strain at 39 and 42°C (see Fig. S4A). In contrast, deletion of *BCY1*, which encodes the regulatory subunit of PKA, had a minimal, nonsignificant effect on the growth of C. auris, while the *bcy1*Δ mutant had less cell density than the WT strain after 10 h of incubation at both 30 and 37°C. Consistent with these results, exogenous addition of cAMP significantly increased PKA activity ∼2.5-fold in the WT strain, whereas basal and induced PKA activity was markedly reduced in *tpk1*Δ and *tpk1*Δ *tpk2*Δ mutants (Fig. 1D). In contrast, deletion of *TPK2* only weakly reduced PKA induction by exogenous cAMP, whereas deletion of *BCY1* significantly enhanced the basal PKA activity (Fig. 1D). Since it is possible that *TPK1* could be upregulated to compensate for absence of *TPK2*, we compared the expression levels of *TPK1* in WT and *tpk2*Δ mutant strains by quantitative reverse transcription-PCR (qRT-PCR). However, we found that *TPK1* expression levels did not change by *TPK2* deletion (see Fig. S4B). Likewise, *TPK2* expression levels did not change by *TPK1* deletion (see Fig. S4B). All of these results suggested that Tpk1 and Tpk2 have major and minor roles, respectively, in growth and PKA catalytic activity, whereas Bcy1 has a negative role.

10.1128/mBio.02729-21.4 FIG S4 Growth analysis of the cAMP/PKA pathway mutants and expression analysis of *TPK1* and *TPK2* in Candida auris. (A) Growth curves of WT (B8441) and each gene deletion mutant were generated at 39 and 42°C in YPD medium. The strains are listed as follows: *cyr1*Δ (YSBA21), *bcy1*Δ (YSBA4), *tpk1*Δ (YSBA13), *tpk2*Δ (YSBA17), and *tpk1*Δ *tpk2*Δ (YSBA24). Each curve represents data from two-independent experiments. The OD_600_ was measured with a multichannel bioreactor (Biosan Laboratories) for 30 to 115 h based on the growth rate. (B) Expression analysis of *TPK1* or *TPK2* in *tpk2*Δ (YSBA17) or *tpk1*Δ (YSBA13) mutants. C. auris WT was used as a control. Cells were cultured overnight at 30°C in liquid YPD medium, subcultured to OD_600_ 0.8, and extracted for total RNA. The expression level of each gene was normalized to that of *ACT1*, and the fold change was calculated relative to the basal expression level of each gene in the WT. Three independent biological experiments with three technical replicates were performed. The data represent means ± SEM. The data were analyzed statistically by the Student’s *t*-test using Prism 8.0 (NS, not significant [*P *>* *0.05]). Download FIG S4, PDF file, 0.3 MB. Copyright © 2021 Kim et al. 2021 Kim et al. https://creativecommons.org/licenses/by/4.0/ This content is distributed under the terms of the Creative Commons Attribution 4.0 International license.

Fungal thermotolerance is known to be regulated by the evolutionarily conserved heat shock transcription factor Hsf1 and heat shock proteins (20). Therefore, we measured the expression level of genes encoding *HSF1* (B9J08_001120) and heat shock proteins (*HSP12*, B9J08_001940; *HSP70*, B9J08_000483; *HSP78*, B9J08_004285; *HSP90*, B9J08_004918; and *HSP104*, B9J08_000476) in the WT and mutant C. auris strains. In all strains, expression of *HSP12*, *HSP70*, and *HSP78* was significantly induced, from 5- to 20-fold, upon a temperature shift from 30 to 42°C (Fig. 1E). Deletion of *CYR1* or *TPK1* and *TPK2* significantly reduced the induction that resulted from the temperature increase (Fig. 1E). This result suggested that the cAMP/PKA pathway is involved in thermotolerance of C. auris and may modulate the expression of diverse heat shock proteins. Notably, although induction of expression of *HSP78* and *HSP90* was greater in the *bcy1*Δ mutant than in the WT strain (Fig. 1E), deletion of *BCY1* did not further increase thermotolerance of C. auris and, indeed, slightly decreased it (Fig. 1A and B). From all of these results, we concluded that balanced modulation of the cAMP/PKA pathway is required for the growth and thermotolerance of C. auris and that Cyr1 is not the only upstream regulator of Tpk1 and Tpk2.

### The role of the cAMP/PKA signaling pathway in the morphological transition of *C. auris*.

**FIG 2:**
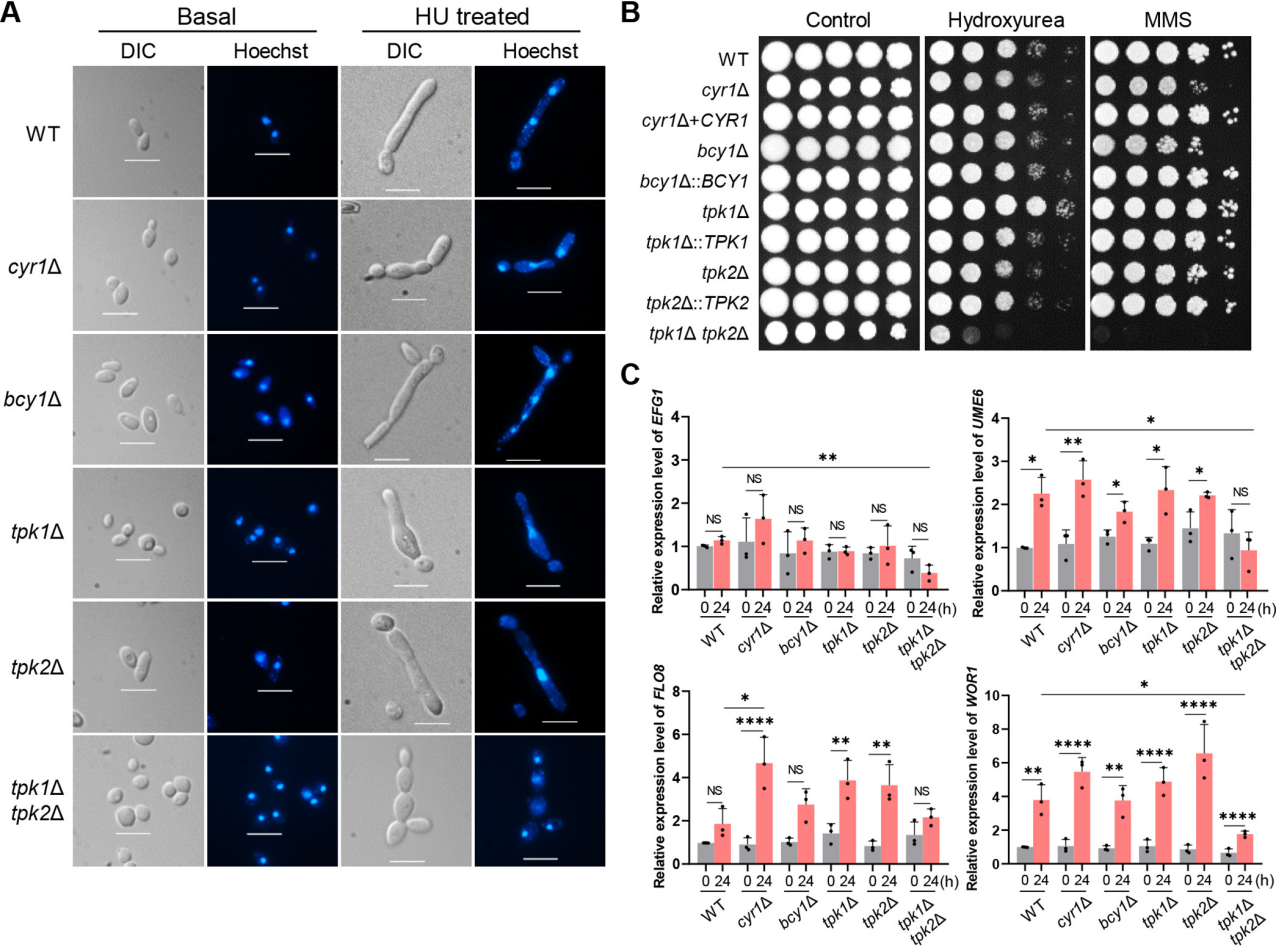
Role of Cyr1 and PKA in filamentous growth of C. auris. (A) Hydroxyurea (HU)-mediated filamentous growth. Cells were cultured overnight at 30°C in YPD medium and subcultured to an OD_600_ of 0.8 in fresh YPD medium. The subcultured cells were resuspended in YPD broth containing 100 mM HU, followed by incubation for 24 h at 30°C. The cells were then fixed in 10% formalin and stained with Hoechst solution. Representative microscopic images of each strain are shown. Scale bars, 10 μm. (B) Spot assays for measuring genotoxic sensitivity. Cells were cultured in liquid YPD medium at 30°C overnight, serially diluted (1 to 10^4^), spotted onto plates supplemented with 100 mM HU or 0.03% methyl methane-sulfonate (MMS), incubated for 3 days at 30°C, and photographed. (C) qRT-PCR analysis of genes involved in morphological switching to the formation of pseudohyphae. Cells were cultured overnight at 30°C in YPD medium, subcultured to OD_600_ 0.8 in fresh YPD medium, and extracted for total RNA (time zero sample). A portion of these were resuspended in media containing 100 mM HU, incubated for another 24 h at 30°C, and extracted for total RNA (24 h sample). The expression levels of *EFG1*, *FLO8*, *WOR1*, and *UME6* were normalized using *ACT1* as the standard, and the fold change was calculated relative to the basal expression level of each gene in the WT. Three independent biological experiments with three technical replicates were performed. (The medium was YPD broth containing 1% yeast extract, 2% peptone, and 2% dextrose; plates contained agar and YPD broth. Data in panel C represent the mean ± SEM. Statistical significance of difference was determined by one-way ANOVA with Bonferroni’s multiple-comparison test (*, *P *<* *0.05; **, *P *<* *0.01; ***, *P *<* *0.001; ****, *P *<* *0.0001; NS, not significant).

The cAMP/PKA pathway also plays a conserved role in fungal morphogenesis (21, 22). In response to certain environmental cues, such as high temperature and blood serum, C. albicans can undergo bud-to-hypha transition, and this ability is an important virulence trait (23–25). In C. auris, agents that impose DNA-damaging stress, such as hydroxyurea (HU) and methyl methane-sulfonate (MMS), can induce pseudohyphal growth (26). In addition, passage through the mammalian body temperature and high salt concentration (10% NaCl) can induce filamentous growth of some C. auris strains (27, 28). This background led us to ask if the cAMP/PKA signaling pathway regulates morphological “switching” of C. auris, from yeasts to pseudohyphae. We used HU to induce the morphological transition in cells of the WT and *cyr1*Δ, *bcy1*Δ, *tpk1*Δ, *tpk2*Δ, and *tpk1*Δ *tpk2*Δ deletion mutants. The cells were cultured in yeast extract-peptone-dextrose (YPD) liquid medium supplemented with 100 mM HU for 24 h. At this time, a large number of WT cells had formed pseudohyphae (Fig. 2A; see also Fig. S5), as has been observed by others (26). The pseudohyphae formed by the *cyr1*Δ, *tpk1*Δ, and *tpk1*Δ *tpk2*Δ mutant cells were shorter than the WT cells (Fig. 2A; see also Fig. S5), and the pseudohyphae were much shorter in the *tpk1*Δ *tpk2*Δ mutant than in the *cyr1*Δ and *tpk1*Δ mutants (Fig. 2A). The *tpk1*Δ *tpk2*Δ mutant cells were still nearly round after 24 h, although there were series of interconnected cells (Fig. 2A). Cells of the *bcy1*Δ and *tpk2*Δ mutants produced pseudohyphae very similar to the WT cells (Fig. 2A; see also Fig. S5). The WT and mutant cells were also tested in the spot assay for sensitivity to MMS and HU. In this test, cells of the *cyr1*Δ, *bcy1*Δ, and *tpk1*Δ *tpk2*Δ mutants had less resistance to MMS or HU than the WT cells, and the *tpk1*Δ mutant had greater resistance to HU than the WT (Fig. 2B).

10.1128/mBio.02729-21.5 FIG S5 Roles of the cAMP/PKA pathway in pseudohypha formation of C. auris. WT and gene deletion mutant cells were cultured overnight at 30°C in YPD medium and subcultured to OD_600_ 0.8 in fresh YPD medium. The subcultured cells were resuspended in YPD broth containing 100 mM HU and then incubated for 24 h at 30°C. The cells were then fixed in 10% formalin and stained with Hoechst solution. Representative microscopic images of each strain are shown. Scale bars, 10 μm. Download FIG S5, PDF file, 0.2 MB. Copyright © 2021 Kim et al. 2021 Kim et al. https://creativecommons.org/licenses/by/4.0/ This content is distributed under the terms of the Creative Commons Attribution 4.0 International license.

In C. albicans, the filamentation and cell elongation is controlled by numerous transcription factors, including Efg1, Flo8, Wor1, and Ume6 (29–33). Therefore, we measured the expression level of *EFG1* (B9J08_002834), *FLO8* (B9J08_000401), *WOR1* (B9J08_004027), and *UME6* (B9J08_000592) in the WT and mutants in response to HU. Expression levels of *EFG1* were unaffected by HU in all strains, except that it was slightly reduced by HU in the *tpk1*Δ *tpk2*Δ mutant (Fig. 2C). In contrast, the expression levels of *UME6* and *WOR1* were significantly enhanced by HU treatment in the WT strain (Fig. 2C). Notably, their expression was normally induced in all mutants like the WT, except for the *tpk1*Δ *tpk2*Δ mutant (Fig. 2C). Expression of *FLO8* was enhanced by HU treatment in the *cyr1*Δ, *tpk1*Δ, and *tpk2*Δ mutants, but not in the WT or in the *tpk1*Δ *tpk2*Δ mutant (Fig. 2C). Based on these results, we suggest that Tpk1 and Tpk2 have major and minor roles, respectively, in regulating the HU-mediated filamentous growth of C. auris, possibly through Ume6 and Wor1, and their regulation is mediated by unknown upstream factors as well as Cyr1.

### Role of cAMP/PKA pathway in stress response and adaptation of *C. auris*.

**FIG 3:**
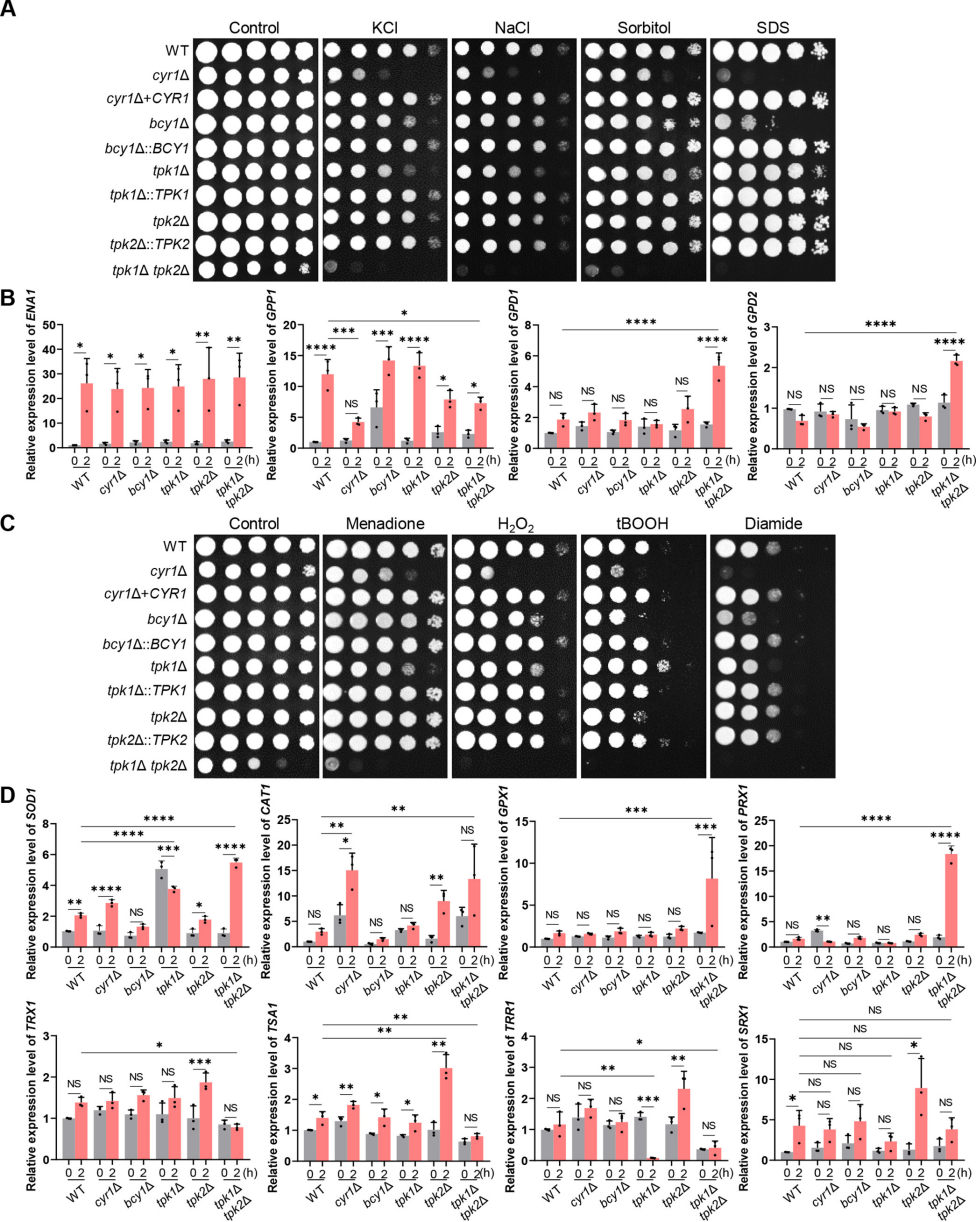
Role of Cyr1 and PKA in osmotic and oxidative stress responses of C. auris. (A) Spot assays for measuring osmotic stress sensitivity. The WT and *cyr1*Δ (YSBA21), *cyr1*+*CYR1* (YSBA38), *bcy1*Δ (YSBA4), *bcy1*Δ::*BCY1* (YSBA29), *tpk1*Δ (YSBA13), *tpk1*Δ::*TPK1* (YSBA36), *tpk2*Δ (YSBA17), *tpk2*Δ::*TPK2* (YSBA26), and *tpk1*Δ *tpk2*Δ (YSBA24) mutant strains were cultured in liquid YPD medium at 30°C overnight; serially diluted (1 to 10^4^); spotted on YPD plates supplemented with 1.5 M KCl, 1.5 M NaCl, 2 M sorbitol, or 0.1% SDS; incubated for 3 days at 30°C; and photographed. A representative image of three independent experiments is shown. In panels B to D, the WT strain and the *cyr1*Δ (YSBA21), *bcy1*Δ (YSBA4), *tpk1*Δ (YSBA13), *tpk2*Δ (YSBA17), and *tpk1*Δ *tpk2*Δ (YSBA24) deletion mutant strains were used. (B) qRT-PCR analysis of the osmotic stress-related genes *ENA1*, *GPP1*, *GPD1*, and *GPD2.* Cells were cultured overnight at 30°C in YPD medium, subcultured to OD_600_ 0.8 in fresh YPD medium, and extracted for total RNA (referred to as the time zero sample). A portion was resuspended in YPD broth containing 1.5 M NaCl, incubated for another 2 h, and extracted for total RNA (2-h sample). (C) Spot assays for measuring oxidative stress sensitivity. Cells were cultured in liquid media at 30°C overnight; serially diluted (1 to 10^4^); spotted onto plates supplemented with 10 mM H_2_O_2_, 1.8 mM *tert-*butyl hydroperoxide (tBOOH), 2.5 mM diamide, or 0.03 mM menadione; incubated for 2 days at 30°C; and photographed. One representative image of three independent experiments is shown. (D) qRT-PCR analysis of the oxidative stress-related genes *SOD1*, *CAT1*, *GPX1*, *TRX1*, *PRX1*, *TSA1*, *TRR1*, and *SRX1*. Cells were cultured overnight at 30°C in liquid media, subcultured to OD_600_ 0.8 in media containing 0.03 mM menadione (for the expression level of *SOD1*) or 10 mM H_2_O_2_ (for the expression level of *CAT1*, *GPX1*, *TRX1*, *PRX1*, *TSA1*, *TRR1*, and *SRX1*), and extracted for total RNA. In both panels B and D, the expression level of each gene was normalized using *ACT1* as the standard, and the fold change was calculated relative to the basal expression level of each gene in the WT. The medium was YPD broth containing 1% yeast extract, 2% peptone, and 2% dextrose. Three independent biological experiments with three technical replicates were performed. The data represent means ± SEM. The statistical significance of difference was determined by one-way ANOVA with Bonferroni’s multiple-comparison test (*, *P *<* *0.05; **, *P*, < 0.01; ***, *P *<* *0.001; ****, *P *<* *0.0001; NS, not significant).

Stress response and adaptation to a changing environment are critical for the survival of fungal species in dynamic natural environments and host niches (34, 35). Candida auris can tolerate higher salt concentrations than other *Candida* species (28, 36). Therefore, we examined the response of the cAMP/PKA pathway mutants to several stresses, beginning with their response to osmotic stress. This was done by imposing stress with either a high concentration of salt (1.5 M NaCl or KCl) and sorbitol (2 M). Cells of all the mutant lines, except for the *tpk2*Δ mutant, were more sensitive to these osmotic agents than were cells of the WT strain (Fig. 3A), and the *tpk1*Δ *tpk2*Δ double mutant was more sensitive than the *cyr1*Δ mutant (Fig. 3A). We concluded that balanced regulation of the cAMP/PKA pathway is critical for osmoadaptation in C. auris. Furthermore, based on the cAMP/PKA mutants’ high sensitivity to sodium dodecyl sulfate (SDS) (Fig. 3A), a membrane-destabilizing detergent, it may be that their markedly increased sensitivity to osmotic agents results from perturbed membrane integrity.

Proteins involved in fungal osmoadaptation include Ena1 (a P-type ATPase sodium pump), Gpp1 (glycerol-3-phosphate phosphatase), and Gpd1/2 (glycerol-3-phosphate dehydrogenases). This led us to assess the expression of the genes encoding these proteins in the C. auris strains. In cells of all the strains, when treated with 1.5 M NaCl, expression of *ENA1* (B9J08_002173) was enhanced ∼25-fold compared to the untreated cells. In WT cells, *GPP1* (B9J08_004378) was enhanced approximately 12-fold compared to the untreated WT cells, while its expression was significantly reduced in response to salt treatment in the *cyr1*Δ and *tpk1*Δ *tpk2*Δ mutants compared to the WT cells (Fig. 3B). We suggest that the cAMP/PKA pathway is involved in the regulation of *GPP1* but not of *ENA1*. Notably, expression of *GPD1* (B9J08_003499) and *GPD2* (B9J08_003684) was unaffected by salt treatment in the WT cells, but expression of both were strongly enhanced in salt-stressed *tpk1*Δ *tpk2*Δ mutant cells compared to those of the unstressed mutant (Fig. 3B). The enhancement was much more pronounced than in any of the other mutant strains. It may be, then, that expression of *GPD1* and *GPD2* responds to osmotic stress and that these proteins act as a compensatory mechanism to restore osmotic balance in the *tpk1*Δ *tpk2*Δ mutant.

We next addressed the role of the cAMP/PKA pathway in resistance of C. auris to oxidative stress. Similar to the mutants’ response to osmotic stress, all of the mutant strains were generally more susceptible to oxidative stress agents (hydrogen peroxides, *tert-*butyl peroxide [tBOOH], menadione [a superoxide anion generator], and diamide [a thiol-specific oxidant]) (Fig. 3C). One exception was that the *tpk1*Δ mutant was more resistant to tBOOH, while the *tpk2*Δ mutant was more sensitive to it, both compared to the sensitivity of the WT (Fig. 3C). This is consistent with the idea that Tpk1 and Tpk2 have opposing roles in defense against organic peroxides.

Several oxidative stress defense systems operate in fungi (37, 38). These include superoxide dismutases (SODs) for converting superoxide anion (O_2_**^·^**^–^) to H_2_O_2_ and catalase, glutathione (GSH)-GSH peroxidase (Gpx), thioredoxin (Trx), peroxiredoxin (Prx), and sulfiredoxin systems for detoxifying inorganic or organic peroxides. We found all of the corresponding orthologs to these proteins in C. auris. Therefore, we next addressed which oxidative defense system is regulated by the cAMP/PKA pathway in C. auris. In response to menadione, expression of *SOD1* (B9J08_001318) was enhanced approximately 2- to 3-fold in WT, *cyr1*Δ, and *tpk2*Δ strains (Fig. 3D). However, basal expression levels of *SOD1* in the *tpk1*Δ mutant and menadione-mediated induction of *SOD1* in the *tpk1*Δ *tpk2*Δ mutant were much greater than in the WT (Fig. 3D). Based on this result, we suggest that Tpk1 and Tpk2 are involved in regulation of the SOD system in C. auris. In response to H_2_O_2_, expression of *CAT1* (B9J08_002298) was weakly enhanced in the WT mutant, but both basal and induced levels of *CAT1* were significantly higher in *cyr1*Δ and *tpk1*Δ *tpk2*Δ mutants compared to the WT (Fig. 3D). From this, we suggest that the catalase system is negatively regulated by the cAMP/PKA pathway or activated as a compensatory mechanism. In contrast, expression of *GPX1* (B9J08_003442) and *PRX1* (B9J08_003437) was not significantly enhanced by H_2_O_2_ in the WT strain or in the *cyr1*Δ, *bcy1*Δ, *tpk1*Δ, and *tpk2*Δ mutants, but strongly, approximately 8- to 18-fold, in the *tpk1*Δ *tpk2*Δ mutant (Fig. 3D). Expression of *TSA1* (B9J08_000645), *TRR1* (B9J08_001090), and *TRX1* (B9J08_000543) was either not enhanced or only weakly so by H_2_O_2_ in the WT and *cyr1*Δ mutant strains. Nonetheless, the enhancement of expression of these genes in the *tpk1*Δ *tpk2*Δ mutant was significantly less than in the WT (Fig. 3D). From this result, we suggest that the Gpx, Prx, and Trx systems are regulated by PKA in a Cyr1-independent manner. Expression of *SRX1* (B9J08_000169) was significantly enhanced (4-fold) by H_2_O_2_ in the WT strain but not significantly induced in *cyr1*Δ, *bcy1*Δ, *tpk1*Δ, and *tpk1*Δ *tpk2*Δ mutants (Fig. 3D). When we considered these data collectively, Cyr1 and PKA play redundant and distinct roles in osmotic and oxidative stress response and adaptation of C. auris.

### Unique role of PKA complex in maintaining cell wall integrity.

**FIG 4:**
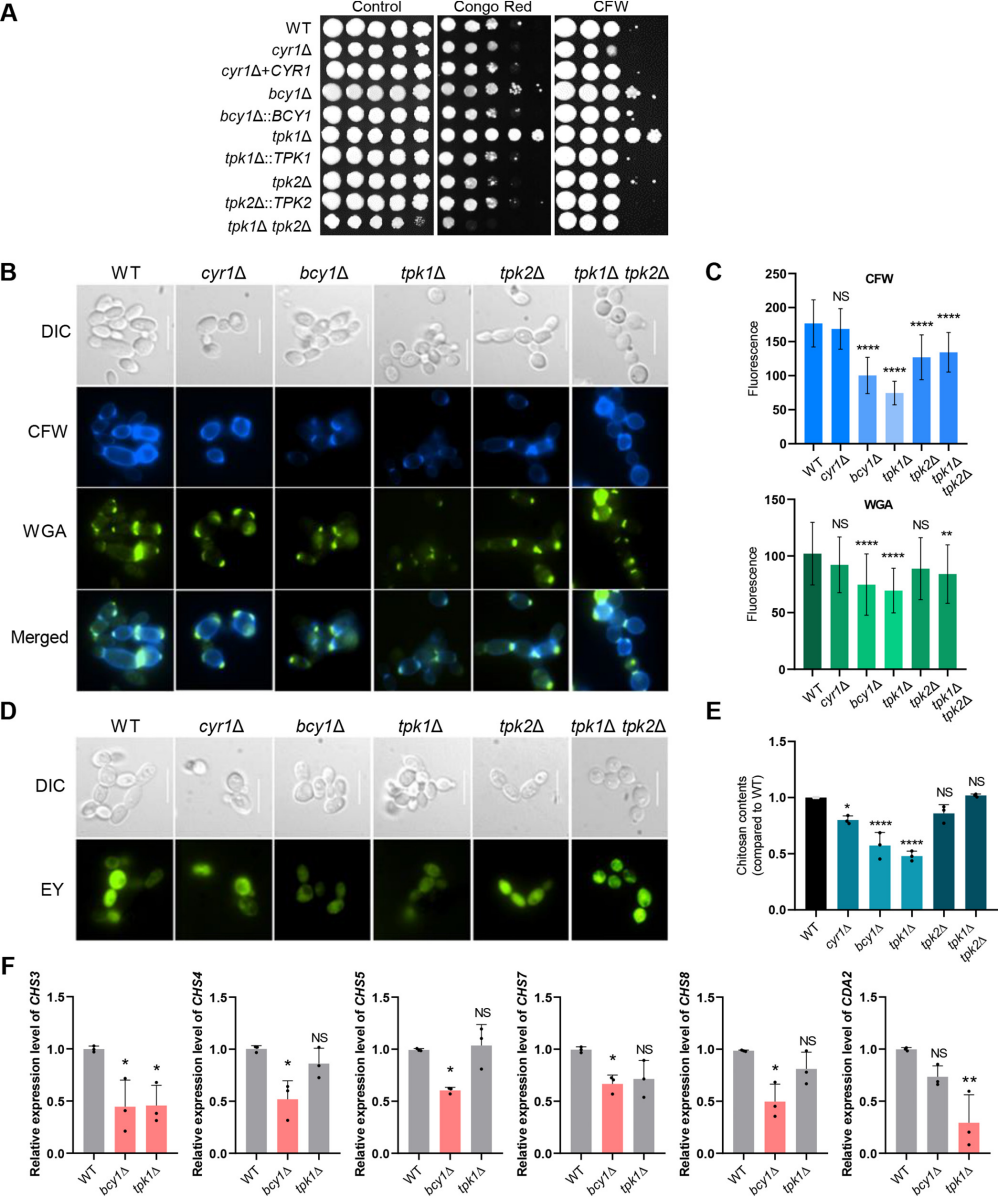
Role of Cyr1 and PKA in maintaining cell wall integrity of C. auris. (A) WT cells or cells of *cyr1*Δ (YSBA21), *cyr1*+*CYR1* (YSBA38), *bcy1*Δ (YSBA4), *bcy1*Δ::*BCY1* (YSBA29), *tpk1*Δ (YSBA13), *tpk1*Δ::*TPK1* (YSBA36), *tpk2*Δ (YSBA17), *tpk2*Δ::*TPK2* (YSBA26), *tpk1*Δ *tpk2*Δ (YSBA24) C. auris strains were cultured in liquid YPD medium at 30°C overnight, serially diluted (1 to 10^4^), spotted on YPD plates supplemented with 0.01% Congo red (CR) or 0.03 mg/ml calcofluor white (CFW), incubated for 3 days at 30°C, and photographed. One representative image of three independent experiments is shown. (B to E) Assessment of cell wall components of the WT and cAMP/PKA pathway *cyr1*Δ (YSBA21), *bcy1*Δ (YSBA4), *tpk1*Δ (YSBA13), *tpk2*Δ (YSBA17), and *tpk1*Δ *tpk2*Δ (YSBA24) deletion mutant strains. (B and C) Chitin staining. Cells were cultured overnight at 30°C in liquid media, subcultured to OD_600_ 0.8, and stained with FITC-conjugated WGA or CFW. In panel B, a representative image of each strain from fluorescence microscopy with the appropriate filters is shown (scale bar, 10 μm). In panel C, quantitative fluorescence measurements of at least 50 individual cells of each strain, measured using ImageJ/Fiji software, are shown. The data represent means ± the standard deviations (SD). The statistical significance of the difference was determined using one-way ANOVA with Turkey’s multiple-comparison test (*, *P *<* *0.05; **, *P *<* *0.01; ***, *P *<* *0.001; ****, *P *<* *0.0001; NS, not significant). (D) Chitosan staining. Cells were cultured overnight at 30°C in liquid media, subcultured to OD_600_ 0.8, stained with EY for chitosan, and observed by fluorescence microscopy. (E) Cells were grown at 30°C in liquid media for 2 days, collected by centrifugation, washed, and used in the MBTH assay for the quantitative measurement of chitosan. Three biological replicates are shown. (F) qRT-PCR analysis of C. auris *CHS3*, *CHS4*, *CHS5*, *CHS7*, *CHS8*, and *CDA2*. WT, *bcy1*Δ (YSBA4), and *tpk1*Δ (YSBA13) strains were cultured overnight at 30°C, subcultured to OD_600_ 0.8, and extracted for total RNA. The expression level of each gene was normalized with *ACT1* as the standard, and the fold change was calculated relative to the basal expression level of fungal cell wall component synthesis-related genes in WT and knockout strains. (The medium was YPD broth containing 1% yeast extract, 2% peptone, and 2% dextrose; the plates contained agar and YPD broth). Three independent biological experiments with three technical replicates were performed. The data represent means ± SEM. The statistical significance of the difference was determined by one-way ANOVA with Bonferroni’s multiple-comparison test (*, *P *<* *0.05; **, *P *<* *0.01; ***, *P *<* *0.001; ****, *P *<* *0.0001; NS, not significant).

We next addressed whether the cAMP/PKA pathway is involved in cell wall integrity maintenance, which could be critical for the survival of C. auris. Chitin and chitosan are fungal cell wall components that, along with glucans and mannoproteins, contribute to resistance of fungi to antifungal drugs (39). We exposed cells of WT C. auris and the deletion mutants to the cell wall-disrupting dyes that target chitin: Congo red (CR) and calcofluor white (CFW). The *tpk1*Δ mutant was much more resistant to the dyes compared to the WT cells, while the *cyr1*Δ mutant was not. Deletion of *TPK2* in the double mutant reduced the resistance of the *tpk1*Δ mutant to the WT level, or even exacerbated it (Fig. 4A). Disruption of *BCY1* also increased resistance to CR and CFW (Fig. 4A) compared to WT cells, perhaps through activation of Tpk2. We concluded from these results that Tpk1 and Tpk2 have opposing roles in regulating chitin synthesis and are independent of Cyr1.

To further verify this finding, we quantitatively measured chitin and chitosan levels in the cAMP/PKA pathway mutants and the WT strain. There was a marked decrease in chitin levels, measured with fluorescence levels in response to CFW and wheat germ agglutinin (WGA), in the *bcy1*Δ and *tpk1*Δ mutants compared to the WT strain and the other mutants (Fig. 4B and C). Similarly, eosin Y (EY) staining revealed that chitosan levels also decreased in the *bcy1*Δ and *tpk1*Δ mutants (Fig. 4D). The 3-methyl-2-benzothiazolinone hydrazone (MBTH) assay, which is used for measuring the fungal cell wall chitosan (40), further confirmed that the *bcy1*Δ and *tpk1*Δ mutants, indeed, had significantly less chitosan than the WT (Fig. 4E). Finally, in agreement with the spot assay data, the chitin and chitosan levels were significantly restored in *tpk1*Δ *tpk2*Δ mutants (Fig. 4B to E) compared to the *tpk1*Δ mutant. The *cyr1*Δ mutant had chitin levels similar to the WT, but slightly lower chitosan levels. We interpret these findings as evidence that the PKA complex, but not Cyr1, mainly regulates cell wall integrity.

To further characterize the role of Bcy1 and Tpk1 in chitin and chitosan biosynthesis, we sought to determine whether expression of the chitin synthase (*CHS*) and chitin deacetylase (*CDA*) genes is regulated by Bcy1 and Tpk1 (Fig. 4F; see also Fig. S6). In fungi, *CHS* and *CDA* are involved in chitin and chitosan biosynthesis (40–44). The following corresponding orthologous proteins are present in C. auris: *CHS1* (B9J08_003879), *CHS2* (B9J08_005077), *CHS3* (B9J08_004150), *CHS4* (B9J08_004972), *CHS5* (B9J08_000868), *CHS6* (B9J08_004541), *CHS7* (B9J08_001816), *CHS8* (B9J08_002856), and *CDA2* (B9J08_004841). Among these, we found that expression levels of *CHS3*, *CHS4*, *CHS5*, *CHS7*, and *CHS8* were significantly less in the *bcy1*Δ mutant than in the WT and that expression of *CHS3* and *CDA2* was less in the *tpk1*Δ mutant than in the WT (Fig. 4F). These findings are further evidence for our suggestion that the PKA complex regulates chitin and chitosan biosynthesis in C. auris.

10.1128/mBio.02729-21.6 FIG S6 Expression analysis of *CHS1*, *CHS2*, and *CHS6* in C. auris by qRT-PCR. WT, *bcy1*Δ (YSBA4), and *tpk1*Δ (YSBA13) strains were cultured overnight at 30°C in YPD medium, subcultured to OD_600_ 0.8, and extracted for total RNA. The expression level of each gene was normalized with that of *ACT1*, and the fold change was calculated relative to the basal expression level of each gene in WT. Three independent biological experiments with three technical replicates were performed. The data represent means ± SEM. The statistical significance of the difference was determined by one-way ANOVA with Bonferroni’s multiple-comparison test (NS, not significant [*P *>* *0.05]). Download FIG S6, PDF file, 0.03 MB. Copyright © 2021 Kim et al. 2021 Kim et al. https://creativecommons.org/licenses/by/4.0/ This content is distributed under the terms of the Creative Commons Attribution 4.0 International license.

### Role of cAMP/PKA pathway in antifungal drug resistance and biofilm formation.

Candida auris is a panresistant fungal species that does not respond to multiple classes of antifungal drugs (2). We sought to address the role of the cAMP/PKA pathway in antifungal drug resistance and began by examining the susceptibility of each mutant to four clinically available antifungal drugs and one fungicide. The antifungal drugs were amphotericin B (AMB), fluconazole (FCZ), 5-fluorocytosine (5-FC), and caspofungin (CSP), and we also used the fungicide fludioxonil.

**FIG 5:**
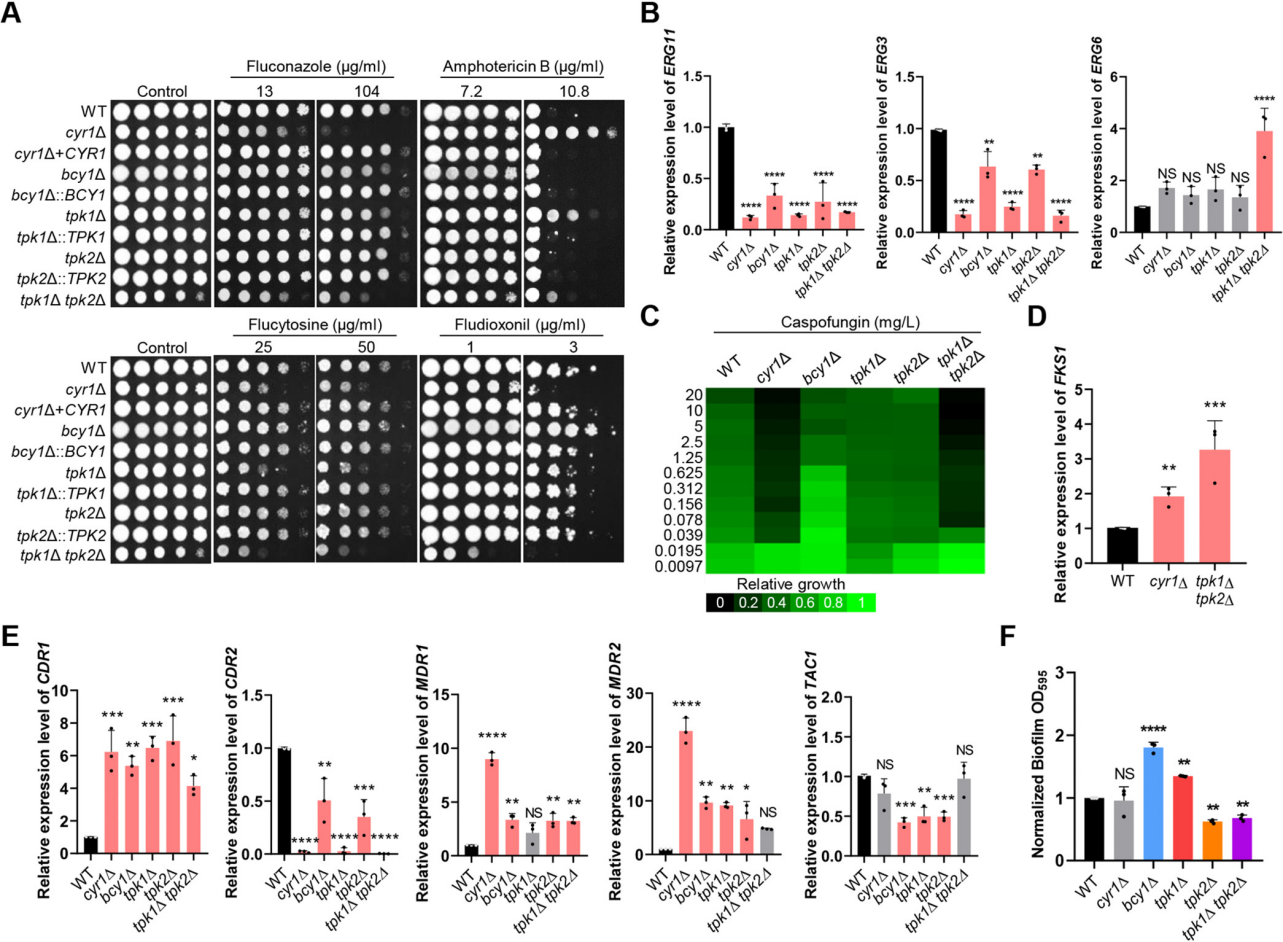
Role of Cyr1 and PKA in resistance to antifungal drugs and biofilm formation of C. auris. (A) Resistance to antifungal drugs. Cells of the WT and mutant *cyr1*Δ (YSBA21), *cyr1*+*CYR1* (YSBA38), *bcy1*Δ (YSBA4), *bcy1*Δ::*BCY1* (YSBA29), *tpk1*Δ (YSBA13), *tpk1*Δ::*TPK1* (YSBA36), *tpk2*Δ (YSBA17), *tpk2*Δ::*TPK2* (YSBA26), and *tpk1*Δ *tpk2*Δ (YSBA24) strains were cultured in liquid YPD medium at 30°C overnight, serially diluted (1 to 10^4^), and spotted onto plates supplemented with the indicated concentrations of amphotericin B, fluconazole, flucytosine, or fludioxonil. The plates were incubated for 3 days at 30°C and photographed. One representative image of three independent experiments is shown. (B) qRT-PCR analysis of the ergosterol synthesis-related genes, *ERG11*, *ERG3*, and *ERG6*, in cells of the WT and *cyr1*Δ (YSBA21), *bcy1*Δ (YSBA4), *tpk1*Δ (YSBA13), *tpk2*Δ (YSBA17), and *tpk1*Δ *tpk2*Δ (YSBA24) mutant strains. Cells were cultured overnight at 30°C in liquid medium, subcultured to OD_600_ 0.8 in fresh YPD medium, and extracted for total RNA. (C) EUCAST MIC test results of caspofungin in the strains from panel B. (D) qRT-PCR analysis of *FKS1* in cells of WT and the mutant *cyr1*Δ (YSBA21) and *tpk1*Δ *tpk2*Δ (YSBA24) strains. Cells were cultured overnight at 30°C in liquid medium, subcultured to OD_600_ 0.8, and extracted for total RNA. Three independent biological experiments with three technical replicates were performed. The data represent means ± SEM. The data were analyzed statistically by Student’s *t*-test using Prism 8.0 (*, *P *<* *0.05; **, *P *<* *0.01; ***, *P *<* *0.001; ****, *P *<* *0.0001; NS, not significant). (E) qRT-PCR analysis of the multidrug resistance-related genes *CDR1*, *CDR2*, *MDR1*, *MDR2*, and *TAC1* in WT cells and in mutant *cyr1*Δ (YSBA21), *bcy1*Δ (YSBA4), *tpk1*Δ (YSBA13), *tpk2*Δ (YSBA17), and *tpk1*Δ *tpk2*Δ (YSBA24) strains. Cells were cultured overnight at 30°C in liquid medium, subcultured to OD_600_ 0.8, and extracted for total RNA. In panels B, D, and E, the expression level of each gene was normalized using *ACT1* as the standard, and the fold change was calculated relative to the basal expression level of each gene in the WT. (F) Biofilm formation assay of C. auris. Cells of the WT and mutant *cyr1*Δ (YSBA21), *bcy1*Δ (YSBA4), *tpk1*Δ (YSBA13), *tpk2*Δ (YSBA17), and *tpk1*Δ *tpk2*Δ (YSBA24) strains were adhered to a polystyrene plate for 90 min. After nonadhered cells were removed, the remaining cells were allowed to form biofilms for 24 h at 37°C in RPMI medium. Biofilm formation was quantified by measuring the OD_595_ of cells that adhered to the bottom of the well after 24 h. For panels B, E, and F, three independent biological experiments with three technical replicates were performed. Data represent means ± SEM. The statistical significance of difference was determined by one-way ANOVA with Bonferroni’s multiple-comparison test (*, *P *<* *0.05; **, *P *<* *0.01; ***, *P *<* *0.001; ****, *P *<* *0.0001; NS, not significant). The medium was YPD broth containing 1% yeast extract, 2% peptone, and 2% dextrose unless noted otherwise.

The *cyr1*Δ mutant was much more susceptible to FCZ and more resistant to AMB than the WT (Fig. 5A). The *tpk1*Δ mutant was also more susceptible to FCZ and more resistant to AMB than the WT, albeit to a lesser extent than the *cyr1*Δ mutant (Fig. 5A). This pattern was not observed for the *tpk2*Δ mutant. Deletion of *BCY1* weakly increased susceptibility to FCZ, but not to AMB (Fig. 5A).

Given these results, we measured the expression level of the gene that encodes lanosterol 14-α demethylase (*ERG11*), which is a target of FCZ. The expression level of *ERG11* was significantly decreased in all deletion mutants compared to the WT (Fig. 5B). Similarly, expression of *ERG3*, encoding C-5 sterol desaturase, was also less in all mutant strains than in the WT strain, but expression of *ERG6*, encoding sterol 24-C-methyltransferase, was significantly upregulated in the *tpk1*Δ *tpk2*Δ mutant only, relative to the WT. We suggest that either inhibition or constitutive activation of the cAMP/PKA pathway perturbs biosynthesis of ergosterol. AMB targets membrane ergosterol so that the low levels of membrane ergosterol levels were likely responsible for AMB resistance that we observed in the *cyr1*Δ and *tpk1*Δ mutants.

Similar to their response to FCZ, the *cyr1*Δ and *tpk1*Δ *tpk2*Δ mutants were much more susceptible to 5-FC, CSP, and fludioxonil than was the WT (Fig. 5A and C). In contrast, the *bcy1*Δ mutant was more resistant to fludioxonil and CSP than the WT strain, although these were responded similarly to 5-FC (Fig. 5A and C). We were interested to find that expression of *FKS1*, which encodes a β-1,3-glucan synthase that is the target of CSP, was significantly upregulated in *cyr1*Δ and *tpk1*Δ *tpk2*Δ mutants compared to the WT (Fig. 5D). From this, we suggest that the cAMP/PKA pathway affects the resistance of the C. auris cells to CSP by influencing *FKS1* expression. We also concluded that Cyr1, Tpk1/Tpk2, and Bcy1 have redundant or distinct roles in antifungal drug susceptibility depending on the specific drug.

Antifungal drug resistance can be also indirectly regulated through overexpression of multidrug transporters and efflux pumps. In C. albicans, the genes *CDR*, *MDR*, and *TAC1* are, respectively, a multidrug transporter of the ABC family, a multidrug efflux pump, and a transcriptional activator of drug-responsive genes (16). Candida auris had the following orthologous proteins to these: *CDR1* (B9J08_000164), *CDR2* (B9J08_002451), *MDR1* (B9J08_003981), *MDR2* (B9J08_000368), and *TAC1* (B9J08_004819). We measured the expression level of *CDR1*, *MDR1*, and *MDR2* in all strains, and observed that, generally, expression was greater in all deletion mutants than in the WT strain (Fig. 5E). In particular, the expression level of *CDR1*, *MDR1*, and *MDR2* was greater in the *cyr1*Δ mutant than in the *tpk1*Δ *tpk2*Δ mutant (Fig. 5E), indicating that Cyr1 has a PKA-independent role in controlling these genes. In contrast to the expression of *CDR1*, *MDR1*, and *MDR2*, the levels of *CDR2* expression were generally lower in the deletion mutants than in the WT strain, but expression of *TAC1* was lower only in the *bcy1*Δ, *tpk1*Δ, and *tpk2*Δ mutants than in the WT (Fig. 5E). Collectively, these data suggested that the cAMP/PKA pathway can modulate expression of drug targets or multidrug transporters and efflux pumps.

In pathogenic fungi, biofilm formation is another major contributing factor to antifungal drug resistance within a host (45, 46). Several studies reported that Tpk1 and Tpk2 play negative and positive roles, respectively, in biofilm formation of C. albicans (47–49). Therefore, we addressed whether the cAMP/PKA pathway regulates biofilm formation in C. auris. Notably, we found that Cyr1 was not involved in biofilm formation, but Tpk1 and Tpk2 play opposite roles (Fig. 5F; see also Fig. S7). The *tpk1*Δ mutant exhibited 1.4-fold increased levels of biofilm, whereas the *tpk2*Δ and *tpk1*Δ *tpk2*Δ mutant showed 2-fold decreased levels of biofilm (Fig. 5F). Deletion of *BCY1* enhanced biofilm formation (1.8-fold) (Fig. 5F). Collectively, these results showed that Tpk1 and Tpk2 are negative and positive regulators, respectively, but Cyr1 is dispensable, for biofilm formation in C. auris.

10.1128/mBio.02729-21.7 FIG S7 Roles of the cAMP/PKA pathway in biofilm formation of C. auris. Each strain was adhered to a polystyrene plate for 90 min in RPMI medium at 37°C, nonadhered cells were removed, and the remaining cells were grown for 24 h at 37°C in RPMI medium. Biofilm formation was quantified by measuring the OD_595_ of cells that adhered to the bottom of the well after 24 h. Three independent biological experiments with three technical replicates were performed. Error bars indicate the SEM. The statistical significance of difference was determined by one-way ANOVA with Bonferroni’s multiple-comparison test (*, *P *< 0.05; **, *P *<* *0.01; ***, *P *<* *0.001; ****, *P *<* *0.0001; NS, not significant). Download FIG S7, PDF file, 0.02 MB. Copyright © 2021 Kim et al. 2021 Kim et al. https://creativecommons.org/licenses/by/4.0/ This content is distributed under the terms of the Creative Commons Attribution 4.0 International license.

### Role of cAMP/PKA pathway in disinfectant resistance.

**FIG 6:**
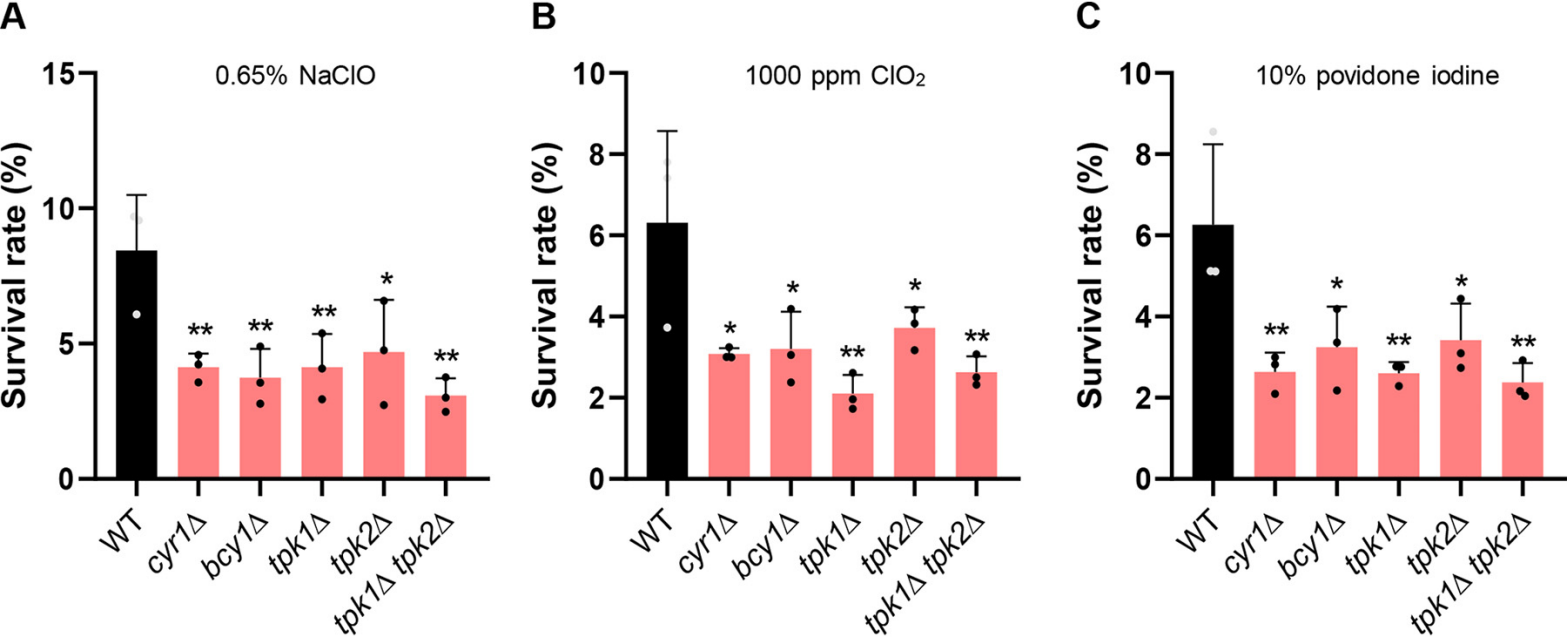
Role of Cyr1 and PKA in disinfectant susceptibility of C. auris. The survival rates of the WT strain and *cyr1*Δ (YSBA21), *bcy1*Δ (YSBA4), *tpk1*Δ (YSBA13), *tpk2*Δ (YSBA17), and *tpk1*Δ *tpk2*Δ (YSBA24) mutant strains were measured after exposure to sodium hypochlorite (0.65% NaClO) (A), chlorine dioxide (1,000 ppm ClO_2_) (B), or 10% povidone-iodine (C) for 30 s as previously described (52). The percent survival rate was calculated by comparing the colony pop-up ratio between the control group and the disinfectant-treated group. Three independent biological experiments were performed; data represent means ± SEM. The statistical significance of the difference was determined by one-way ANOVA with Bonferroni’s multiple-comparison test (*, *P *<* *0.05; **, *P *<* *0.01; ***, *P *<* *0.001; ****, *P *<* *0.0001; NS, not significant).

Candida auris can survive very long time in health care environments and can be transmitted readily between patients (50, 51). For this reason, appropriate disinfection process is critical for limiting nosocomial infection of C. auris in a hospital environment. Therefore, we examined here how modulation of the cAMP/PKA pathway affects the susceptibility of C. auris to chlorine-based and iodine-based disinfectant, which are known to be relatively effective against the pathogen (52, 53). When treated for 30 s with chlorine-based disinfectants (0.65% NaClO or 1,000 ppm ClO_2_) or iodine-based disinfectant (10% povidone-iodine), approximately 5 to 10% of C. albicans WT strain survived (Fig. 6). Notably, we found that all cAMP/PKA mutants (*cyr1*Δ, *bcy1*Δ, *tpk1*Δ, *tpk2*Δ, and *tpk1*Δ *tpk2*Δ) showed significantly increased (∼2-fold) susceptibility to the disinfectants compared to WT (Fig. 6). Collectively, these data showed that either inhibition or activation of the cAMP/PKA pathway can increase the disinfectant susceptibility of C. auris.

### Role of cAMP/PKA pathway in the transition of *C. auris* cells from a haploid to a diploid form.

**FIG 7:**
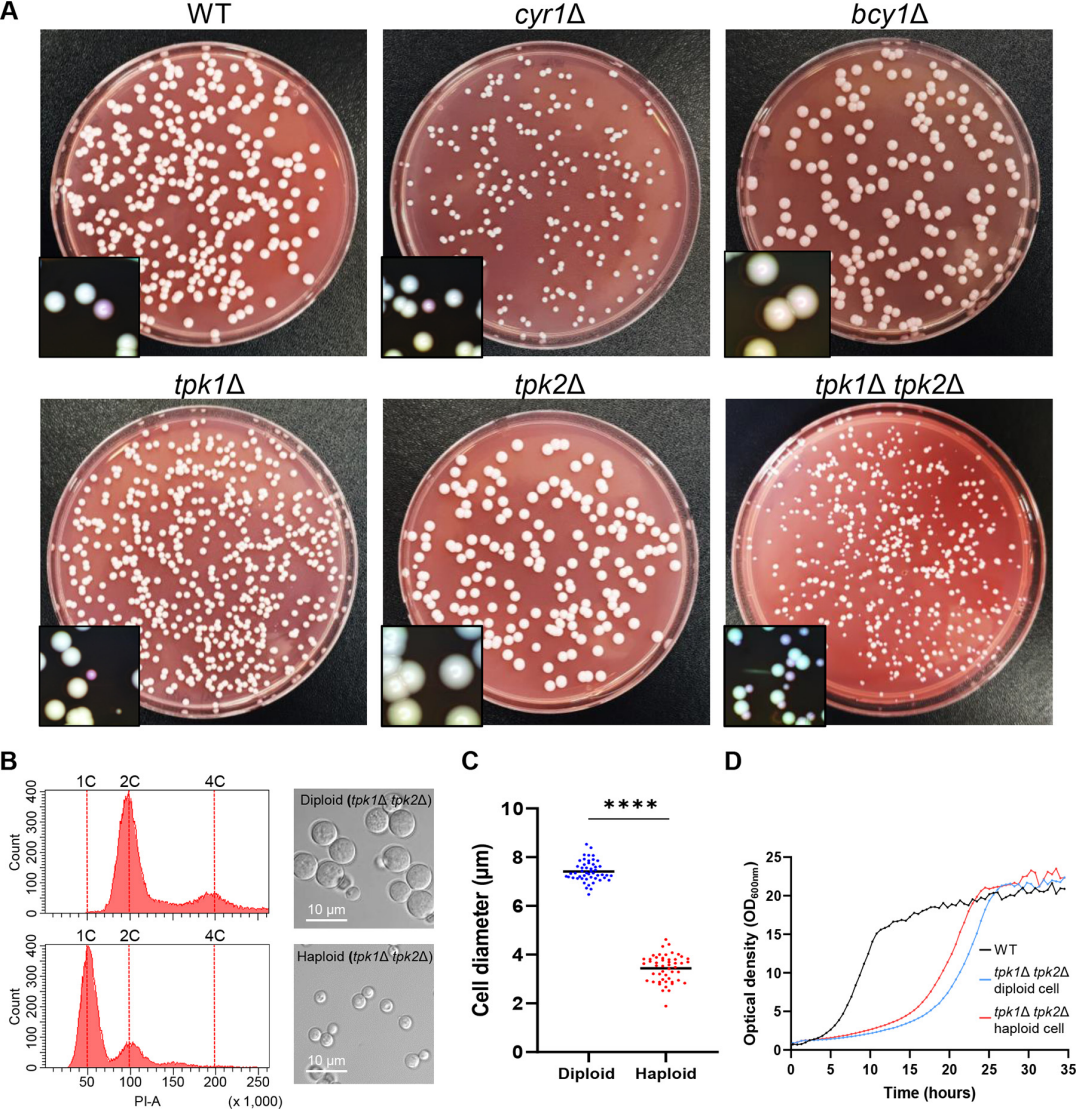
Unique role of PKA in ploidy switch of C. auris. (A) Assessment of ploidy-switching cells from the WT or the *cyr1*Δ (YSBA21), *bcy1*Δ (YSBA4), *tpk1*Δ (YSBA13), *tpk2*Δ (YSBA17), and *tpk1*Δ *tpk2*Δ (YSBA24) mutant strains. Approximately 100 to 200 cells were plated on YPD medium containing 5 μg/ml phloxine B, incubated at 25°C for 5 to 7 days, and photographed. A representative image from two independent experiments is shown. (B) Morphological and DNA content analysis of haploid and diploid cells from *tpk1*Δ *tpk2*Δ mutants. The haploid and diploid cells of the *tpk1*Δ *tpk2*Δ mutant recovered from panel A were cultured in liquid YPD medium at 30°C for 48 h, fixed, photographed, and subjected to FACS. Scale bar, 10 μm. (C) Average cell diameter of *tpk1*Δ *tpk2*Δ diploid and haploid cells. The diameter of at least 50 individual cells of each strain was measured with ImageJ/Fiji software. The data represent means ± the SD. The data were analyzed using Student’s *t*-test using Prism 8.0 (****, *P *<* *0.0001). (D) Growth rate of cells of the WT and the *tpk1*Δ *tpk2*Δ mutant strain. Diploid cells are blue, and haploid cells are red. Cells were incubated for 30 h at 30°C, and their cell densities were measured at OD_600_ by a multichannel bioreactor (Biosan Laboratories). This growth curve is representative of two independent experiments. (The medium was YPD broth containing 1% yeast extract, 2% peptone, and 2% dextrose; the plates contained agar and YPD broth.)

Recently, it has been reported that C. auris in all clades can undergo a ploidy switch from haploid to diploid (54). Diploid cells grew more slowly but were larger and more virulent than the haploid cells (54). Considering the role of the cAMP/PKA pathway in morphological “switching” to pseudohyphae formation (Fig. 2A), we sought to determine whether the cAMP/PKA pathway is involved in the ploidy switching. We first measured the frequency of ploidy shifting in all strains by plating each strain on solid YPD medium containing phloxine B. In this situation, the colonies will appear white or pink, depending on whether they consist of haploid or diploid cells, respectively (54). A small percentage (approximately 0.2%) of WT cells converted to diploid after 5 to 7 days of incubation (Fig. 7A); this result is consistent with the previous report. We observed about the same number of pink colonies on plates with the *cyr1*Δ and *tpk1*Δ mutants as we did for the WT strain (Fig. 7A) and no pink colonies on plates with the *bcy1*Δ and *tpk2*Δ mutants. We observed a strikingly large number of small pink colonies, approximately 53% of all colonies, on plates with the *tpk1*Δ *tpk2*Δ mutant (Fig. 7A). Based on this, we suggest that Tpk1 and Tpk2 play redundant roles in the ploidy “switching” in C. auris. Fluorescence-activated cell sorting (FACS) analysis confirmed that the white and pink colonies of the *tpk1*Δ *tpk2*Δ mutant represent haploid and diploid cells, respectively (Fig. 7B). Also consistent with accurate detection of the ploidy, the diameters of the putative diploid cells of the *tpk1*Δ *tpk2*Δ mutant were twice those of haploid cells (Fig. 7B and C), and the diploid cells of the *tpk1*Δ *tpk2*Δ mutant grew more slowly than the haploid (Fig. 7D). Collectively, we interpret these data as evidence that the PKA complex, but not Cyr1, is a critical regulator of ploidy switching of C. auris.

### Role of cAMP/PKA pathway in the virulence of *C. auris*.

**FIG 8:**
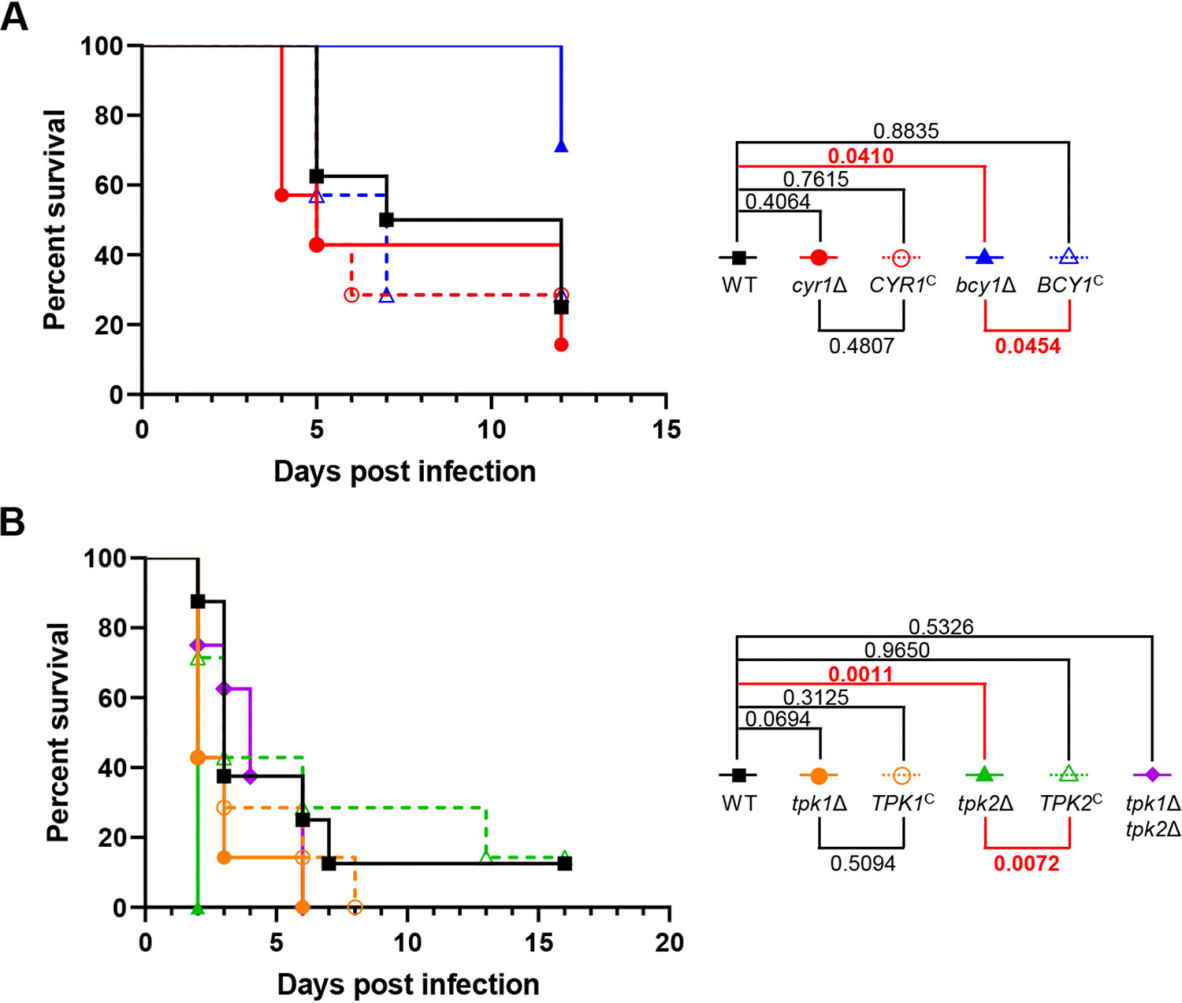
Role of Cyr1 and PKA in the virulence of C. auris. Seven-week-old female A/J mice were injected intravenously with 1 × 10^7^ cells of strains of C. auris. (A) Mice were injected with cells of WT (B8441), *cyr1*Δ (YSBA21), *cyr1*Δ+*CYR1* (YSBA38), *bcy1*Δ (YSBA4), or *bcy1*Δ::*BCY1* (YSBA29) strains. (B) Mice were injected with cells of WT, *tpk1*Δ (YSBA13), *tpk1*Δ::*TPK1* (YSBA36), *tpk2*Δ (YSBA17), *tpk2*Δ::*TPK2* (YSBA26), or *tpk1*Δ *tpk2*Δ (YSBA24) strains. Statistical analysis was performed with a log-rank (Mantel-Cox) test using Prism 8.0.

Finally, we determined whether the cAMP/PKA pathway is required for the pathogenicity of C. auris. To this end, we established the murine systemic infection model with A/J mice and intravenous injection of C. auris B8441 cells. First, we tested the virulence of C. auris B8441 by intravenously injecting the 10^7^ to 2 × 10^8^ WT cells. Inoculating A/J mice with of 5 × 10^7^ to 2 × 10^8^ C. auris B8441 cells can be lethal by 2 days postinfection and inoculating 10^7^ cells can be lethal by 5 days postinfection (see Fig. S8). So that we could detect hypo- or hypervirulence of the cAMP/PKA mutants, we set the intravenous inoculum concentration at 10^7^ cells per mouse. Unexpectedly, the *cyr1*Δ and *tpk1*Δ *tpk2*Δ mutants, which exhibited *in vitro* growth defects at 37°C, exhibited WT levels of virulence (Fig. 8A and B). Notably, however, the *bcy1*Δ mutant was less virulent than the WT and its complemented strain (Fig. 8A). In contrast, the *tpk2*Δ mutant was hypervirulent (Fig. 8B). The data are consistent with the suggestion that constitutive activation of the cAMP/PKA pathway, but not its inhibition, attenuates the virulence of C. auris.

10.1128/mBio.02729-21.8 FIG S8 Survival rate of A/J mice infected with various inocula of C. auris WT strain B8441. Seven-week-old A/J female mice (five mice per group) were infected intravenously with the indicated inocula of C. auris WT cells (B8441). The cells were cultured overnight in YPD medium at 30°C, washed three times with distilled water, and resuspended in PBS for intravenous infection. Download FIG S8, PDF file, 0.02 MB. Copyright © 2021 Kim et al. 2021 Kim et al. https://creativecommons.org/licenses/by/4.0/ This content is distributed under the terms of the Creative Commons Attribution 4.0 International license.

## DISCUSSION

The pan-resistant C. auris has only recently emerged as a fungal pathogen of global importance, and consequently, its pathogenicity-related signaling pathways are still largely unknown. We focused on the cAMP/PKA pathway as one of the most well-known signaling pathways in fungal pathogenicity and identified its major signaling components and their pathobiological functions in C. auris. These components include adenylyl cyclase, Cyr1, and the subunits of PKA—the catalytic subunits Tpk1 and Tpk2 and the regulatory subunit Bcy1.

**FIG 9:**
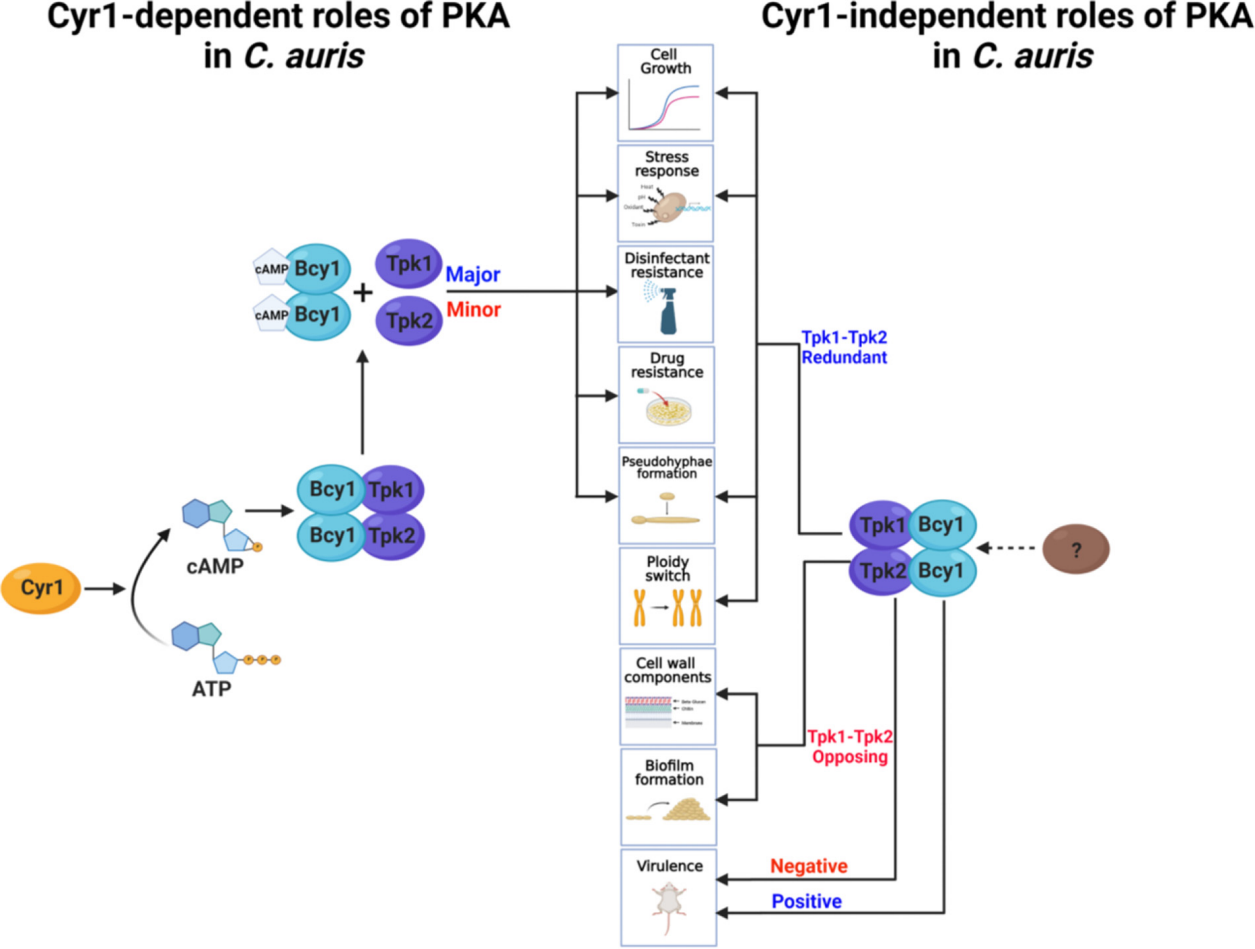
Proposed pathobiological functions and regulatory mechanisms of the cAMP/PKA pathway in C. auris. A single adenylyl cyclase, Cyr1, catalyzes the conversion of ATP to cAMP. The cAMP binds to and releases the regulatory subunit of PKA, namely, Bcy1, from its two PKA catalytic subunits, Tpk1 and Tpk2. These have major and minor roles in pathogenicity, respectively. The activated PKA is involved in (i) cell growth at various temperatures, (ii) response and adaptation to stress, (iii) resistance to antifungal drugs, and (iii) the morphological transition of single cells to pseudohyphae. In addition, PKA can contribute to cell growth, response and adaptation to stress, and morphological transition in a Cyr1-independent manner. In particular, PKA plays unique roles in the transition of cells from haploid to diploid, in chitin and chitosan biosynthesis, and in biofilm formation in a Cyr1-independent manner. Most notably, Bcy1 and Tpk2, but not Cyr1 and Tpk1, have positive and negative roles, respectively, in the virulence of C. auris.

We have demonstrated here that the cAMP/PKA pathway plays pleiotropic roles in C. auris, including growth, morphological transitions, response and adaptation to stress, control of its cell wall integrity, changes in ploidy from haploid to diploid, resistance to antifungal drugs and disinfectants, biofilm formation, and pathogenicity. We highlight two unique findings in this study. First, PKA has pathobiological functions that are both dependent and independent of Cyr1. Second, hyperactivation of the cAMP/PKA pathway attenuates the virulence of C. auris, but inhibition of the pathway does not (Fig. 9). These findings are rather unexpected considering the known functions of the cAMP/PKA pathway in other fungal pathogens.

Based on our finding that the *cyr1*Δ mutant exhibited severe growth defects at 30 to 42°C, it appears that maintaining basal cAMP levels is critical for normal growth of C. auris. This is also supported by the evidence that, in S. cerevisiae, Cyr1, an adenylate cyclase that is the only source of intracellular cAMP, is an essential protein. Its conditional mutations confer slow growth, increased accumulation of glycogen, and increased sensitivity to heat and oxidative stress (55). The importance of basal cAMP levels is also supported by the observation that the *tpk1*Δ *tpk2*Δ *tpk3*Δ triple mutant is lethal in the budding yeast. In contrast, Cyr1 orthologs are not essential, but are important, for the growth of ascomycetous fungal pathogens including C. albicans, Aspergillus flavus, and A. fumigatus: null mutations of adenylyl cyclase genes (C. albicans *CDC35*, A. flavus *acyA*, and A. fumigatus *acyA*) were not catastrophic, but markedly hampered normal growth (56–58). Notably, however, deletion of the adenylate cyclase Cac1 or deletion of both genes for the catalytic subunits of PKA (*PKA1* and *PKA2*) in the basidiomycetous fungal pathogen C. neoformans does not affect cell viability and growth rate at all (59). Although basal levels of cAMP appear to be critical in C. auris, it also appears that this requirement differs between fungal species.

The unique features that we have identified in the C. auris cAMP/PKA pathway is that PKA has Cyr1-dependent and -independent functions in diverse pathobiological processes and that basal PKA activity is required for fungal growth regardless of Cyr1 activity. Growth of *TPK1* or *TPK2* deletion mutants of C. albicans have normal growth, but the *tpk1/tpk1 tpk2/tpk2* double mutant grows extremely slowly, similar to the *cdc35/cdc35* mutant (60). It may be that the two isoforms of the PKA catalytic subunit have a redundant role in growth of C. albicans, mainly downstream of Cdc35. In C. neoformans, the *cac1*Δ mutant also phenocopies the *pka1*Δ *pka2*Δ mutant (61). In C. auris, we concluded that Tpk1 and Tpk2 are major and minor catalytic subunits, respectively, of PKA. Therefore, it seems reasonable that growth was affected in the *tpk1*Δ and in the *cyr1*Δ mutant, but not in the *tpk2*Δ mutant. However, the *tpk1*Δ *tpk2*Δ mutant exhibited more severe defects in growth, thermotolerance, stress responses, and morphological transitions to pseudohypha formation than either the single mutant or the *cyr1*Δ mutant. It may be that Tpk1 and Tpk2 are synergistic and modulated by other upstream factors in a Cyr1-independent manner.

The Cyr1-independent unique role of Tpk1 and Tpk2 was most evident in the transition from haploid to diploid in C. auris. It is remarkable that only the *tpk1*Δ *tpk2*Δ double mutant, not the single mutant or the *cyr1*Δ mutant, exhibited the dramatic increased transition to the diploid condition compared to the WT (more than 50% of the cells became diploid). Ploidy variation is a common characteristic of fungal pathogens, providing genetic diversity and serving as a driving force for evolution (62). In addition, ploidy changes affect cell size, which may also affect adaptation to environmental changes, stress responses, and resistance to antifungal drugs (63, 64). In C. neoformans, PKA activity is involved in the formation of atypically large, polyploid titan cells (65, 66). The formation of these titan cells results from enhanced PKA expression or loss of regulation of the PKA regulatory subunit by *PKR1.* In titan cells, the capsule, an unusual feature of this fungus, is larger than in typical cells and the cell wall is altered. In C. auris, however, when both *TPK1* and *TPK2* were removed, in other words, when PKA activity was absent, the cells transitioned to diploid and were larger. These observations lead us to suggest that a role of PKA activity in cell ploidy is conserved in some fungi, but its role may differ among them.

The broad resistance of C. auris (panresistance) to antifungal drugs has led to its global importance. In particular, C. auris is markedly resistant to the azole family (7, 67, 68), more so than most fungi. In this study, we found that the cAMP/PKA pathway was critically important in drug resistance, controlling expression of drug target genes. These are, for example, *ERG11* for azoles, *FKS1* for echinocandins, and transporter and efflux pump genes that are affected by numerous drugs. Furthermore, we demonstrated that PKA, but not Cyr1, regulates biofilm formation, which may contribute to the *in vivo* resistance of C. auris to antifungal drugs. In C. albicans, deletion of *RAS1* or *CYR1* increases its susceptibility to azole family drugs and upregulates *CDR1* (17), evidence in support of our finding that, in C. auris, *CYR1* deletion markedly increased azole susceptibility and *CDR1* expression. Also, in C. albicans, Tpk1 and Tpk2 were negative and positive regulators, respectively, of biofilm formation (47, 49); this also confirms our similar results in C. auris. Notably, we found that Cyr1 is not involved in biofilm formation of C. auris. Therefore, the role of the cAMP/PKA pathway in regulation of ergosterol biosynthesis, multidrug transporters, and biofilm formation appears to be conserved in *Candida* species. We showed that deletion of both *TPK1* and *TPK2* dramatically increased the susceptibility of C. auris to fluconazole, flucytosine, caspofungin, and fludioxonil. It may be, then, that Tpk1/Tpk2 inhibitors will prove useful potentiators for enhancing the antifungal activity of the currently available antifungal drugs or fungicides. Furthermore, our finding that mutation of *CYR1*, *TPK1*, *TPK2*, or *BCY1* significantly promoted the disinfectant susceptibility of C. auris suggests that addition of inhibitors or activators of the cAMP/PKA pathway to commercially available disinfectant could further prevent nosocomial infection of C. auris in a health care environment. This exciting possibility will be investigated in the future.

The most unexpected findings made by this study are that inhibition of the cAMP/PKA pathway did not attenuate the virulence of C. auris and that hyperactivation of the PKA complex reduced virulence, although the C. auris cAMP/PKA mutants we constructed may result in different virulence outcome in other infection models. This is surprising for two reasons. First, deletion of adenylyl cyclase or PKA catalytic subunits generally abolishes or highly attenuates the virulence of most animal and plant fungal pathogens, including C. albicans, C. neoformans, A. fumigatus, Magnaporthe grisea, and Fusarium graminearum (56, 69–74). Second, both C. auris *cyr1*Δ and *tpk1*Δ *tpk2*Δ mutants not only had severely disrupted growth at 37°C, but also exhibited increased sensitivity to stress. It may be that the increased haploid-to-diploid switching frequency of the *tpk1*Δ *tpk2*Δ mutant contributes to the virulence of C. auris by compensating for its retarded cell growth; it has been shown that diploid C. auris cells are more virulent than haploid ones (54). It is unclear why the virulence of the *bcy1*Δ mutant was attenuated. Although increased stress sensitivity of the *bcy1*Δ mutant may contribute to its reduced virulence, similar phenotypes were also found in *cyr1*Δ and *tpk1*Δ *tpk2*Δ mutants. One possible explanation could be based on the finding that the *bcy1*Δ mutant reached a lower level of cell density than that of the WT after 10 h of incubation at 37°C, although the *cyr1*Δ and *tpk1*Δ *tpk2*Δ mutants eventually reached the WT cell density after prolonged incubation at 37°C. Bcy1-dependent effector proteins required for the virulence of C. auris should be a focus of future studies. Furthermore, it will be interesting to determine whether we can make a similar finding in other clades of C. auris.

In conclusion, we have demonstrated here that the cAMP/PKA pathway is involved in diverse pathobiological functions, including growth, stress resistance, pseudohypha formation, drug and disinfectant resistance, cell wall integrity, biofilm formation, ploidy switching, and virulence of C. auris. For treatment of C. auris infection, we propose that inhibitors of the cAMP/PKA pathway show potential for use in combination with some currently available antifungal drugs, such as FCZ, CSP, and 5-FC. Activators of the pathway may also be effective when used by themselves. We propose the following topics for future research: (i) characterization of potential Cyr1 upstream regulators, such as a G-protein-coupled receptor or heterotrimeric or small GTP-binding proteins; (ii) identification of the Cyr1-independent PKA upstream regulators; and (iii) elucidation of Cyr1 and PKA-dependent downstream signaling networks. Answering these research question may provide comprehensive understanding of the role of the cAMP/PKA pathway in pan-antifungal drug resistance and pathogenicity of C. auris.

## MATERIALS AND METHODS

### Ethics statement.

Care of animals and all experiments were conducted in accordance with the ethical guidelines of the Institutional Animal Care and Use Committee of Yonsei University, and this committee approved all of the vertebrate studies.

### *C. auris* strains and growth media.

Candida auris strains used in this study are listed in Table S1 in the supplemental material. The parental WT strain, strain B8441 (AR0387) was obtained from the CDC. These isolates and the constructed mutant strains were stored as frozen stocks in 20% glycerol at –80°C. Yeast strains were grown at 30°C with shaking at 200 rpm in 1% yeast extract–2% peptone–2% d-glucose (YPD broth) or on plates containing 2% agar in YPD broth (YPD plates).

10.1128/mBio.02729-21.9 TABLE S1 C. auris strains used in this study. Download Table S1, DOCX file, 0.02 MB. Copyright © 2021 Kim et al. 2021 Kim et al. https://creativecommons.org/licenses/by/4.0/ This content is distributed under the terms of the Creative Commons Attribution 4.0 International license.

### Gene deletion and complementation.

All gene deletion mutants were constructed by using the nourseothricin resistance marker (*CaNAT*) or hygromycin B resistance marker (*CaHYG*) (75) flanked by 0.6- to 1.0-kb 5′ and 3′ regions of each target gene: *CYR1* (B9J08_004540), *BCY1* (B9J08_002818), *TPK1* (B9J08_004030), and *TPK2* (B9J08_002788). The gene deletion mutants *bcy1*Δ and *tpk1*Δ were generated with Cas9-ribonuleotideprotein-mediated transformation. Each gene disruption cassette containing either *CaNAT* or *CaHYG* selection marker was constructed by double-joint PCR (76). To amplify the flanking regions of a target gene, L1-L2 and R1-R2 primer pairs were used in the first round of PCR. The *CaNAT* selection marker was amplified by PCR using the plasmid pV1025 containing the *CaNAT* gene as a template and the primer pairs listed in Table S2 in the supplemental material. The first round of PCR products of the flanking regions and *CaNAT* marker were purified together and used as the templates for the second round of double-joint PCR. In the second round of PCR, 5′- and 3′-gene disruption cassettes containing split *CaNAT* selection markers were amplified by L1-split primer 2 and R2-split primer 1, respectively (see Table S2 in the supplemental material). To set up CRISPR-Cas9, CTG-clade specific CaCas9 and single-guide RNAs were amplified by PCR from plasmid pV1025 and pV1090, respectively (77). For transformation of C. auris with gene disruption cassettes, a previous electroporation protocol was used with modifications (78, 79). Briefly, cells were cultured overnight at 30°C in 50 ml of YPD broth with shaking. Cells were centrifuged, washed once with dH_2_O, resuspended in electroporation buffer (100 mM lithium acetate, 10 mM Tris, 1 mM EDTA [pH 7.5]), and incubated at 25°C with shaking for 1 h. Next, electrocompetent cells were centrifuged, washed once each with dH_2_O and 1 M sorbitol, and finally resuspended in 10 ml of 1 M sorbitol. Approximately 1 μg of disruption cassette dissolved in 5 μl dH_2_O was mixed with 40 μl of electrocompetent cells. Electroporation was performed using the C. albicans protocol on a GenePulsar Xcell (Bio-Rad). Cells were then allowed to recover in YPD broth at 30°C in a shaking incubator for 2 h, plated onto selective YPD agar medium containing 400 μg/ml nourseothricin or 1.8 mg/ml hygromycin B, and further incubated at 30°C for 2 to 3 days. The desired genotype of each positive nourseothricin or hygromycin B-resistant transformant was confirmed by diagnostic PCR and Southern blotting (see Fig. S2). To confirm the phenotypes of the *cyr1*Δ, *bcy1*Δ, *tpk1*Δ, and *tpk2*Δ mutants, corresponding complemented strains, in which each WT allele was either ectopically integrated (*cyr1*Δ+*CYR1*) or reintegrated into its native locus (*bcy1*Δ::*BCY1*, *tpk1*Δ::*TPK1*, and *tpk2*Δ::*TPK2*), were constructed as follows. First, each full-length gene fragment was amplified by Phusion-PCR by using genomic DNA of the WT B8441 strain as a template and each primer pair listed in Table S2 in the supplemental material. The amplified fragments were directly cloned into the TOPO vector (Invitrogen), generating the plasmid pTOP-CYR1, pTOP-BCY1, pTOP-TPK1, pTOP-TPK2. After we confirmed the target sequence, we subcloned the *CYR1*, *BCY1*, *TPK1*, and *TPK2* inserts into the plasmid pYM70 to produce the plasmids pHYG-CYR1, pHYG-BCY1, pHYG-TPK1, and pHYG-TPK2. For the targeted reintegration into its native locus, pHYG-CYR1, pHYG-BCY1, pHYG-TPK1, and pHYG-TPK2 were linearized by HpaI, AvrII, BglII, and AfeI, respectively, and introduced into each mutant by electroporation. The correct genotype of the complemented strain was confirmed by diagnostic PCR (see Fig. S3).

10.1128/mBio.02729-21.10 TABLE S2 Primers used in this study. Download Table S2, DOCX file, 0.02 MB. Copyright © 2021 Kim et al. 2021 Kim et al. https://creativecommons.org/licenses/by/4.0/ This content is distributed under the terms of the Creative Commons Attribution 4.0 International license.

### Total RNA preparation and quantitative RT-PCR.

WT and cAMP/PKA mutant strains of C. auris were inoculated into 50 ml of YPD broth and cultured overnight at 30°C in a shaking incubator. Cells were subcultured in 50 ml of fresh YPD broth until the optical density at 600 nm (OD_600_) was 0.6 to 0.8. At this point, the cells were collected by centrifugation, frozen in liquid nitrogen, and lyophilized. For stress response experiments, 10 ml was sampled for the zero time-point (basal condition) and the remaining 30 ml was further incubated with indicated stress agents or conditions. Total RNA was isolated by the TRIzol extraction method using Easy-Blue (Intron). Complementary DNA (cDNA) was synthesized by reverse transcriptase (Thermo Scientific) with purified total RNA and subsequently used for quantitative PCR using specific primer pairs for each gene with the CFX96 real-time system (Bio-Rad). Expression of *ACT1* served as the control for normalization. Statistical differences between samples were analyzed by one-way analysis of variance (ANOVA) using Bonferroni’s multiple-comparison test. All experiments were performed in triplicate from three biological replicates.

### Growth and stress sensitivity spot assay.

To analyze the growth and sensitivity to various stresses of WT and cAMP/PKA mutant strains of C. auris. To do this, C. auris cells grown overnight at 30°C were serially diluted 10-fold four times (final dilution, 1:10^4^) and then spotted onto YPD plates. For growth measurements, plates were incubated at 30, 37, 39, and 42°C. Growth was assessed qualitatively by photographing the plates 1 to 2 days later.

Various stresses were imposed by adding to the media chemical agents that would impose stress to the cells. Osmotic stress was supplied as sorbitol. Cation and salt stress were imposed with NaCl or KCl; these were supplied in either glucose-rich YPD or in glucose-deficient media (YPD without dextrose). Oxidative stress was supplied with hydrogen peroxide, *tert*-butyl hydroperoxide (an organic peroxide), menadione (a superoxide anion generator), or diamide (a thiol-specific oxidant). Membrane-destabilizing stress was imposed using SDS, and cell wall-destabilizing stress was imposed using CFW and CR, fludioxonil, fluconazole, amphotericin B, or flucytosine for antifungal drug susceptibility. Cells were incubated at 30°C and photographed 1 to 6 days after treatment.

### PKA activity assay.

Cells of WT and cAMP/PKA mutant strains of C. auris were grown overnight to their stationary phase at 30°C in 50 ml of YPD broth. Cells were pelleted, resuspended in 1 ml of 10 mM sodium phosphate buffer (1 mM EGTA, 1 mM EDTA, 10 mM β-mercaptoethanol, 1 mM phenylmethylsulfonyl fluoride, and protease inhibitor cocktail), and lysed by disruption with glass beads at 4°C, and the cell debris was pelleted by centrifugation for 20 min. The supernatant was used for the PKA activity assay. The PKA assay was performed either in the presence or absence of 10 μM cAMP using a PKA kinase activity kit (EZNO).

### Assessment of the morphological transition from yeasts to pseudohyphae.

To induce pseudohypha formation in WT and cAMP/PKA mutant strains of C. auris, cells were grown for 24 h in YPD broth containing 100 mM HU (26). After induction of hypha formation, each sample was fixed in 10% formalin and stained with 10 μg/ml Hoechst 33342 (Thermo Fisher) for microscopy. The fixed, stained samples were incubated in the dark for 0.5 to 1 h and photographed.

### Measurement of chitin and chitosan content in cell walls.

To measure chitin and chitosan content of cells of WT and cAMP/PKA mutant strains of C. auris, cells were cultured overnight in a shaking incubator at 30°C and then pelleted, washed, and resuspended in pH 7.5 phosphate-buffered saline (PBS). Next, cells were stained with 100 μg/ml fluorescein isothiocyanate (FITC)-conjugated WGA, 25 μg/ml CFW, or 30 μg/ml EY for 30 min in the dark. Stained cells were observed and photographed by fluorescence microscopy (Olympus BX51). The fluorescence of at least 50 individual cells was measured with ImageJ/Fiji software.

The chitosan content of the WT and mutant cells was measured quantitatively with a 3-methyl-2-benzothiazolinone hydrazone (MBTH)-based chemical assay (80, 81). Cells were cultured in 50 ml of liquid YPD in a shaking incubator for 2 days at 30°C, collected by centrifugation, washed three times with PBS (pH 7.5), frozen in liquid nitrogen, and lyophilized. After weighing, the lyophilized cells were resuspended in 6% KOH, incubated at 80°C for 30 min with occasional tapping to eliminate nonspecific MBTH-reactive molecules, washed three times with PBS (pH 7.5) to neutralize the pH, and resuspended in PBS to a final concentration of 10 mg/ml. We used 0.1 ml of this preparation in the MBTH assay. For the assay, samples were mixed with 1 M HCl for a final concentration of 0.5 M HCl. Samples were tapped, heated at 110°C for 2 h, transferred to a 25°C water bath for cooling, and 2.5% NaNO_2_ was added to each. The samples were incubated at room temperature for 15 min; 200 μl of 12.5% ammonium sulfamate was added to each sample, which were then incubated for another 5 min at room temperature. Next, 200 μl of 0.25% MBTH was added to each sample, followed by a 30-min incubation at 37°C, the addition of 200 μl of 0.5% FeCl_3_, and a further 5 min of incubation at 37°C. Finally, the optical density of each sample was measured at 595 nm.

### EUCAST MIC test.

WT and mutant C. auris strain cells were cultured overnight at 30°C in YPD medium, washed twice with H_2_O, and then resuspended in H_2_O. For the EUCAST (European Committee on Antimicrobial Susceptibility Testing) MIC test, we used a cell concentration corresponding to an OD_600_ of 1.0. We added 100 μl of the cell suspension to 10 ml of morpholinepropanesulfonic acid (MOPS)-buffered RPMI 1640 media (pH 7.4 with 0.165 M MOPS and 2% glucose for EUCAST) and loaded into 96-well plates containing 2-fold serially diluted drugs. The 96-well plates were incubated at 35°C for 2 days, and the cell density in each well was measured at OD_595_ (82, 83).

### Biofilm assay.

The ability of C. auris to form biofilms was measured as previously described, with modifications (84). Cells of WT and cAMP/PKA mutant strains of C. auris were cultured overnight in 2 ml of YPD broth at 30°C. For this assay, cells were suspended in RPMI 1640 media at a final concentration corresponding to OD_600_ of 0.5, and 200 μl of the suspension was added to flat-bottom 96-well plates. The plates were then sealed with Breathe-Easy sealing membranes to reduce evaporation and to prevent cross-contamination between wells. Plates were incubated at 37°C for 90 min with shaking at 250 rpm, after which the RPMI medium was aspirated, and the wells were washed with 200 μl of PBS, followed by the addition of 200 μl of fresh RPMI 1640 medium. The plates were then resealed and incubated at 37°C for 24 h with shaking at 250 rpm. After 24 h, the RPMI 1640 medium was aspirated from the wells, and the OD_595_ was read on a plate reader. The average density of adhered cells was calculated from the optical density readings from three independent wells in the plate. Data were normalized as follows. The OD_595_ reading of an average blank well was subtracted from the OD_595_ reading of each experimental and control well. The blank-subtracted OD_595_ value of each experimental or control well was then normalized to the mean blank-subtracted OD_595_ for the relevant control wells.

### Disinfectant susceptibility assay.

To quantitatively assess fungicidal activity of chlorine-based disinfectants (0.65% NaClO and 1,000 ppm ClO_2_) and iodine-based disinfectant (10% povidone iodine) against C. auris WT and cAMP/PKA mutants, we followed the protocol of the European Standard EN 13624:2013 with modifications. Briefly, 10^6^ cells in 100 μl of 0.3% bovine albumin solution was added in 900 μl of each disinfectant. After the 30 s of contact time, 100 μl of the resulting suspension was transferred to an e-tube containing 900 μl of appropriate neutralizing solution (Dey/Engley neutralizing solution for chlorine-based disinfectants and double-strength Dey/Engley solution for the iodine-based disinfectant). After 5 min of neutralization, 3 μl of cell suspension was spread on the YPD medium to count the CFU of each strain. As a control group, cells treated only with 0.3% bovine albumin solution without disinfectant treatment were spread on the YPD medium to count the CFU of each strain. The percent survival rate of each strain was calculated by comparing the colony pop-up ratio between the control group and the disinfectant-treated group.

### FACS analysis.

Cells from WT and cAMP/PKA mutant strains of C. auris from a single colony were inoculated into liquid YPD medium, grown at 30°C overnight, harvested, and washed with PBS. Cells were counted, and 10^6^ cells were fixed with 70% ethanol for 14 to 16 h at 4°C, washed twice with PBS, treated with RNase A (200 μg/ml) for 1 h at 37°C, and then washed twice with PBS. The cells were stained in 500 μl of 100-μg/ml propidium iodide for 30 min at room temperature, washed with PBS, and resuspended in PBS. These cells were used for DNA content analysis. In each experiment, at least 30,000 cells were used for the flow cytometry analysis.

### Virulence study.

The *in vivo* virulence of WT and cAMP/PKA mutant strains of C. auris were examined in a murine model. The fungal cells were incubated overnight at 30°C in YPD broth and washed three times with PBS. Cell concentrations were measured and adjusted to 10^8^ cells/ml in PBS. To confirm the CFU and the viability of the inoculum, the diluted cells were plated onto YPD agar plate and incubated at 37°C for 24 h. Six-week-old female mice of the A/J strain were acquired for this study (Japan SLC, Inc.). After arrival, the mice were habituated for 1 week. The fungal cells were injected intravenously as follows: mice were restrained, and their tails were placed in warm (40°C) water to expand the lateral veins. The cell suspension (100 μl) was injected. Survival was monitored daily. A 10% decrease of a mouse’s body weight or abnormal behavior (tilting or whining) was deemed a humane endpoint to the experiment, and the mouse was sacrificed. Statistical analysis was performed with log-rank (Mantel-Cox) test for the murine survival curve.

### Data availability.

We will provide any strain and materials used in this study upon request.
