# Supplementary figures and images for: Adenylyl Cyclase and Protein Kinase A Play Redundant and Distinct Roles in Growth, Differentiation, Antifungal Drug Resistance, and Pathogenicity of Candida auris
Source: mBio. 2021 Oct 19;12(5):e02729-21. doi: 10.1128/mBio.02729-21 (PMC8524339; doi:10.1128/mBio.02729-21)

Figure S1 (Kim et al.)

A

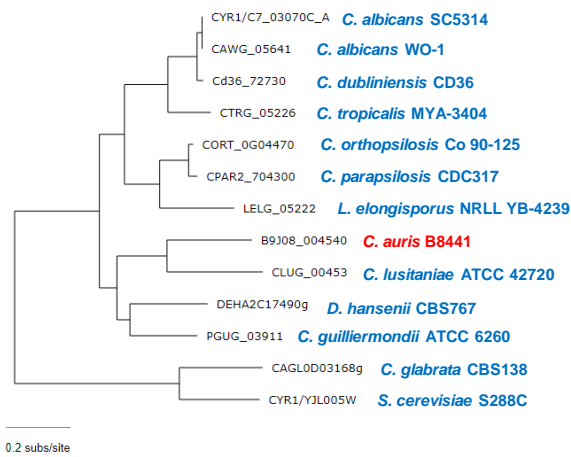

B

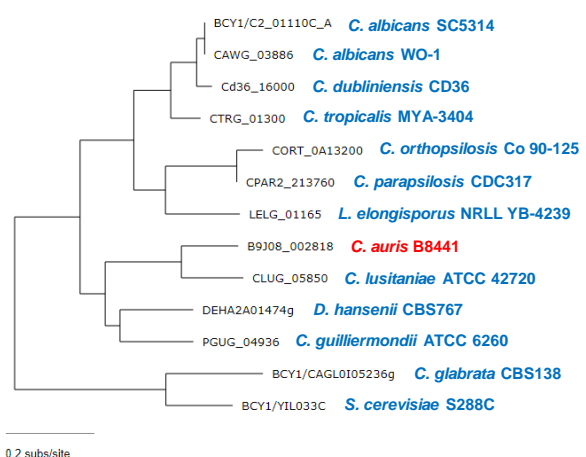

C

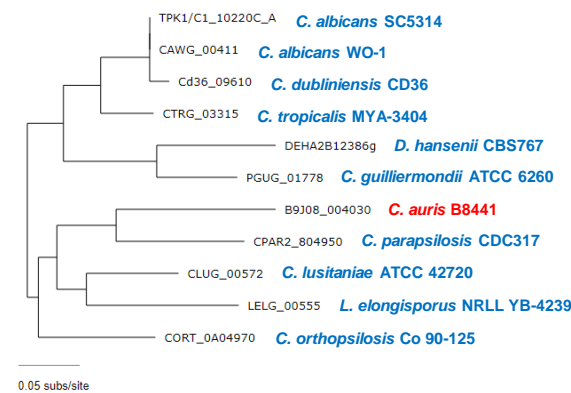

D

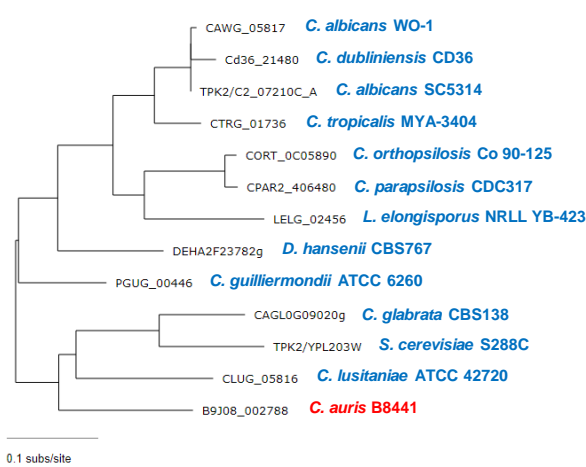

Supplement: FIG S1 [file mbio.02729-21-sf001.pdf]

Figure S2 (Kim et al.)

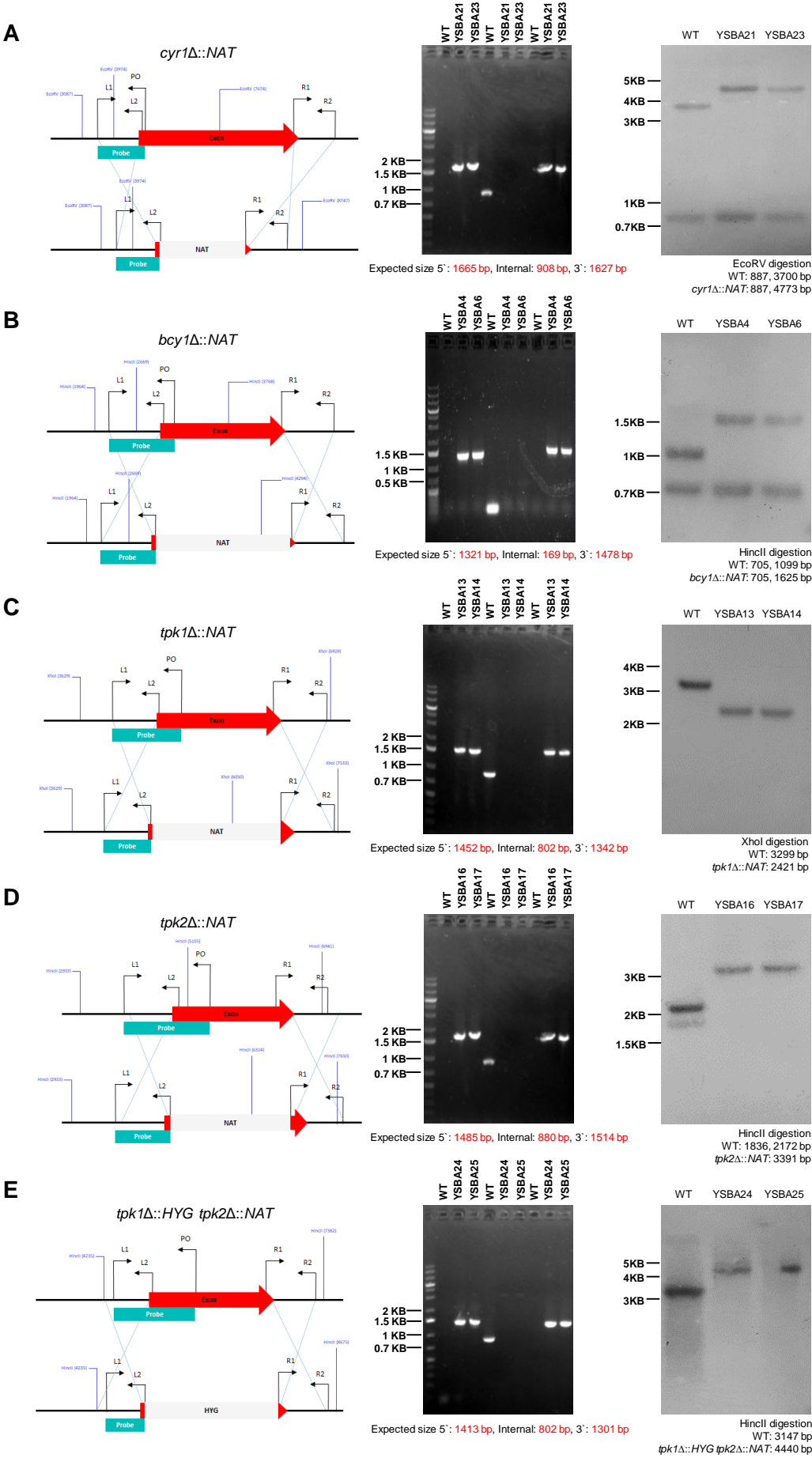

Supplement: FIG S2 [file mbio.02729-21-sf002.pdf]

Figure S3 (Kim et al.)

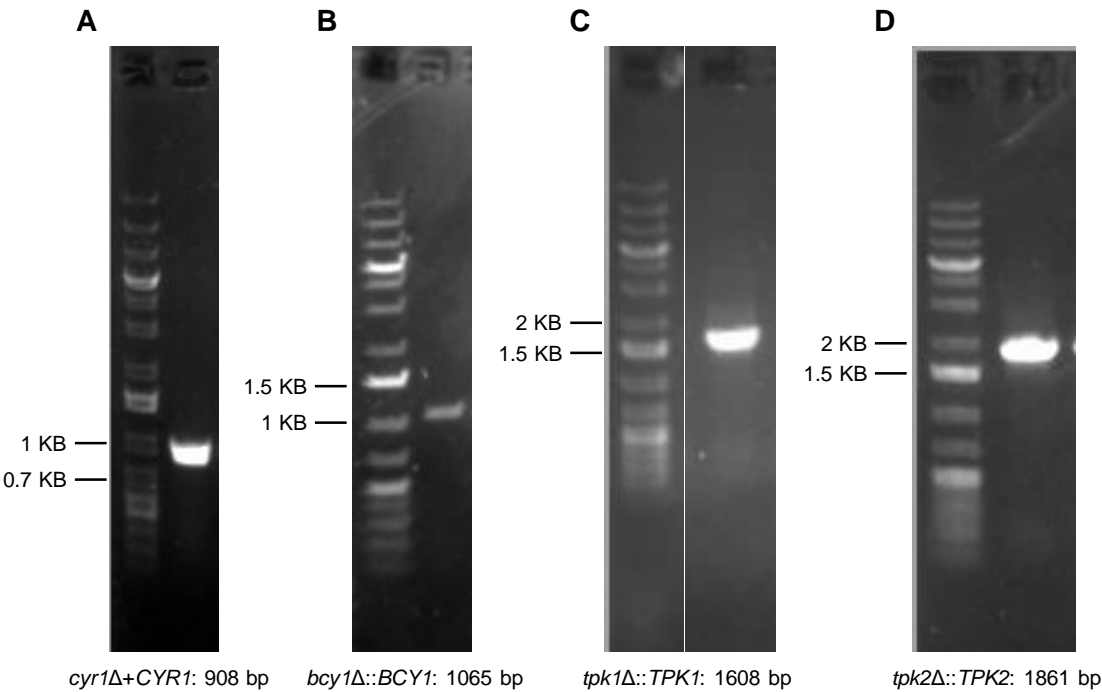

Supplement: FIG S3 [file mbio.02729-21-sf003.pdf]

Figure S4 (Kim et al.)

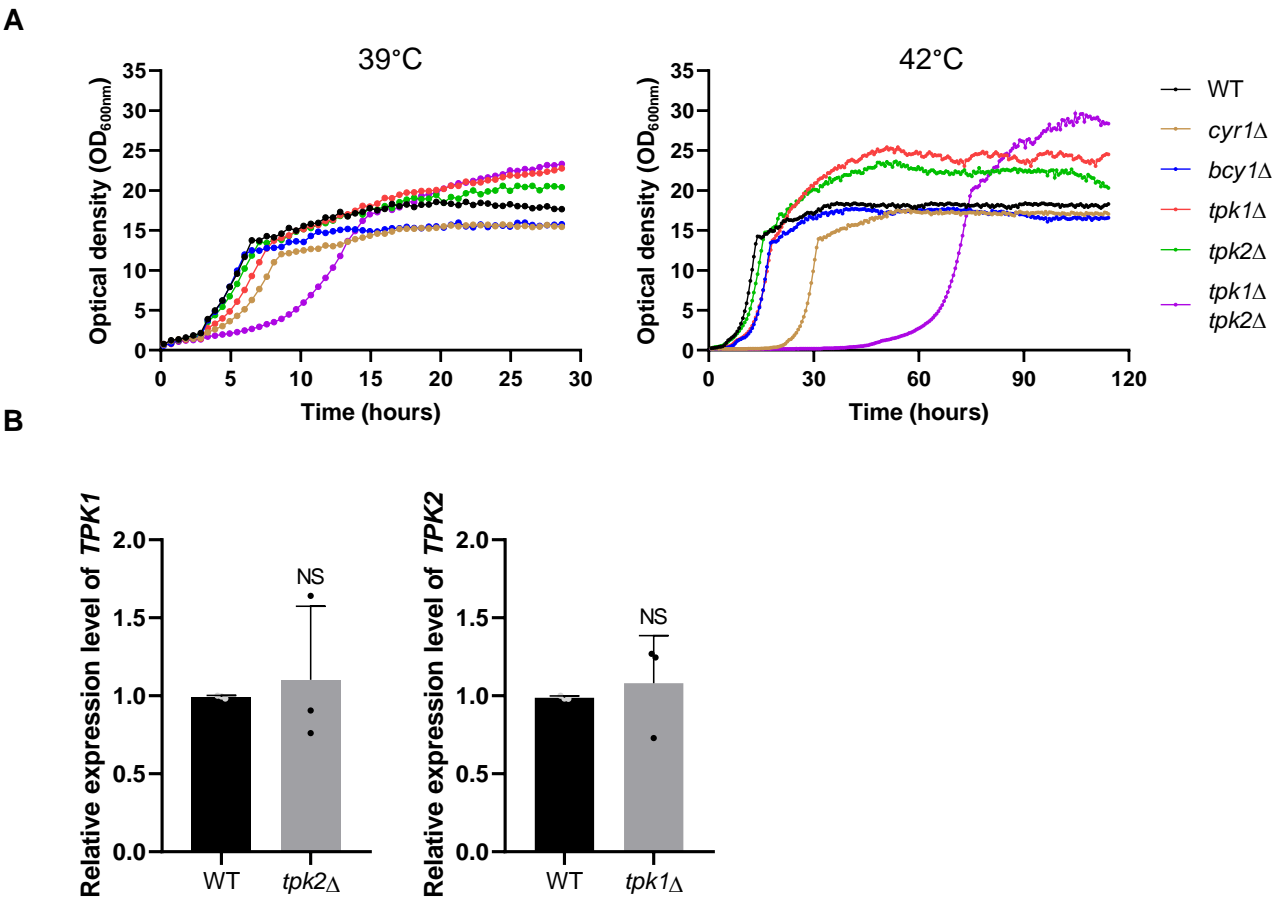

Supplement: FIG S4 [file mbio.02729-21-sf004.pdf]

Figure S5 (Kim et al.)

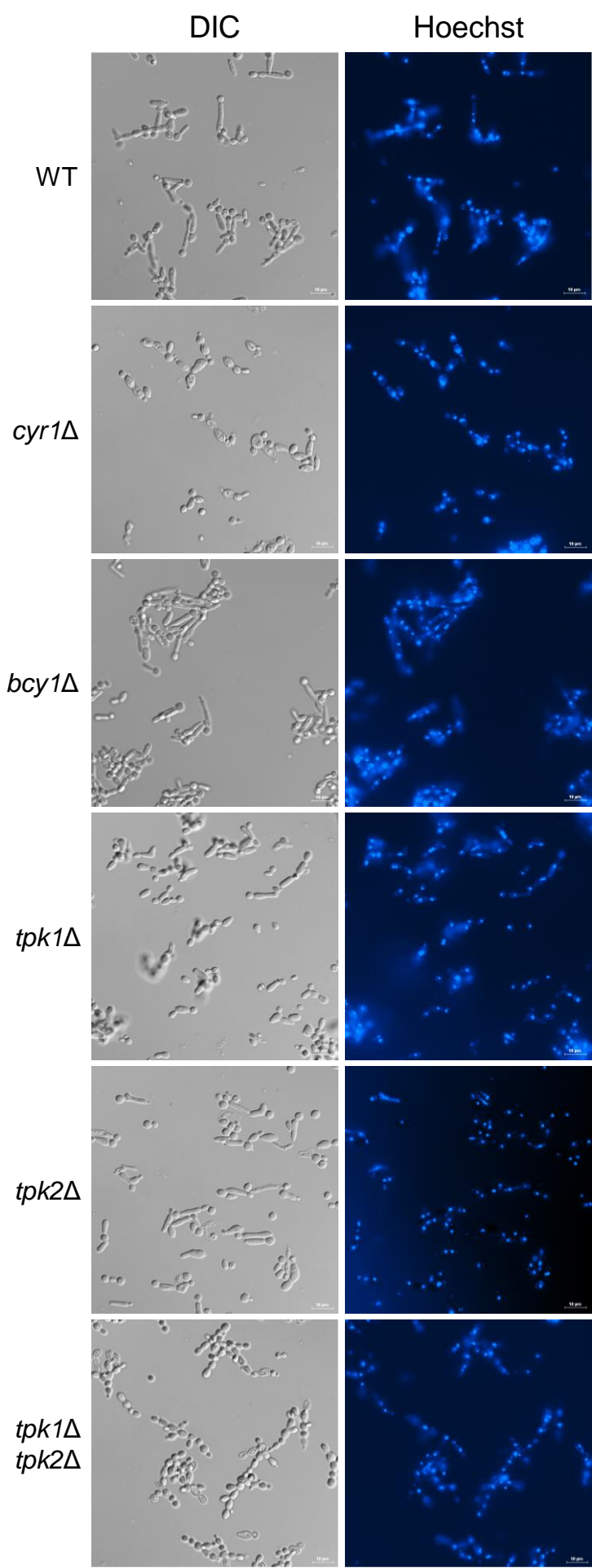

Supplement: FIG S5 [file mbio.02729-21-sf005.pdf]

Figure S6 (Kim et al.)

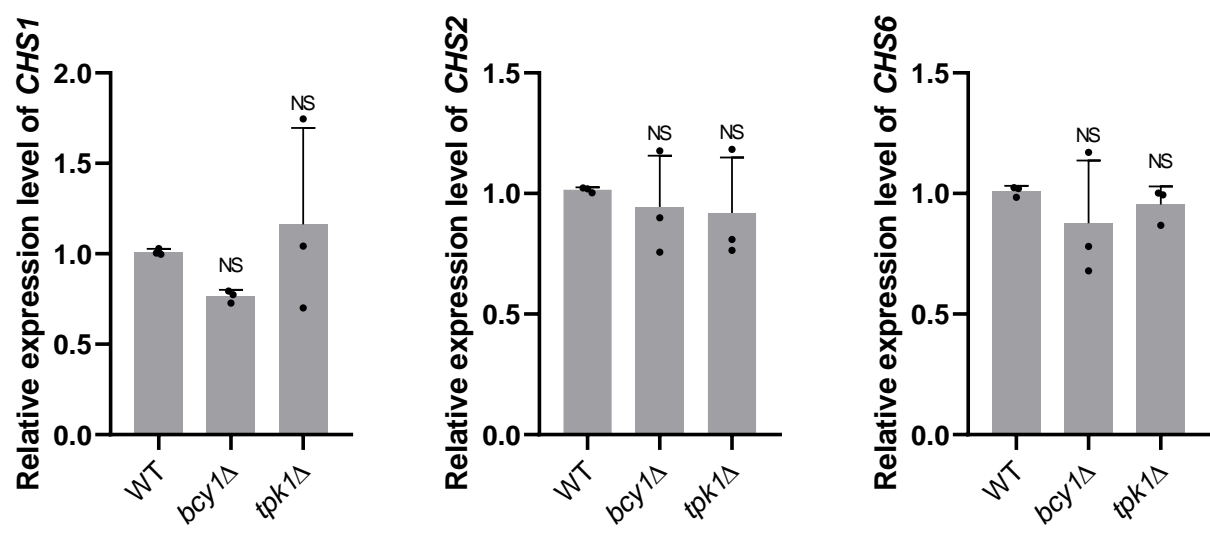

Supplement: FIG S6 [file mbio.02729-21-sf006.pdf]

Figure S7 (Kim et al.)

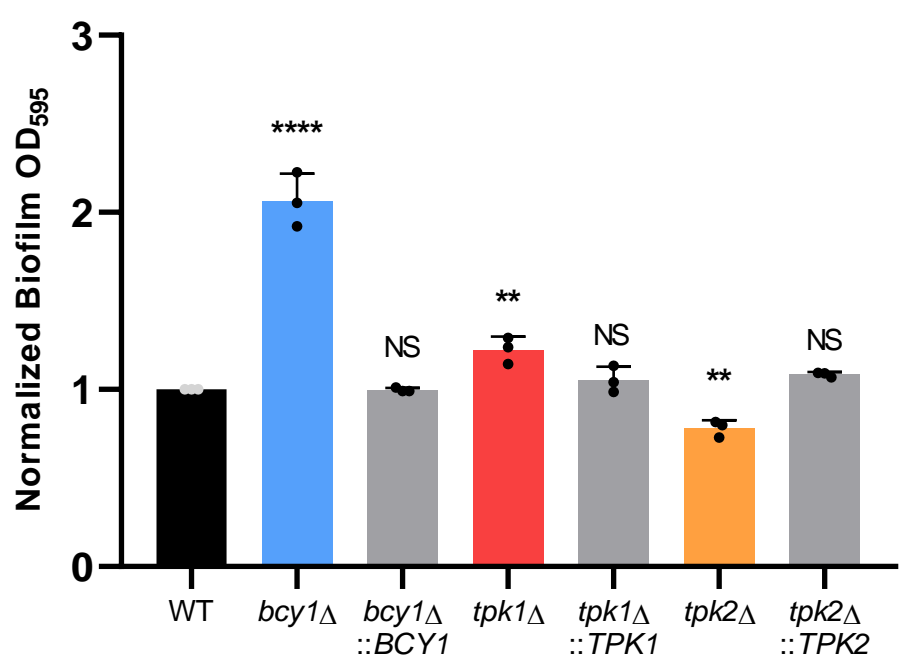

Supplement: FIG S7 [file mbio.02729-21-sf007.pdf]

Figure S8 (Kim et al.)

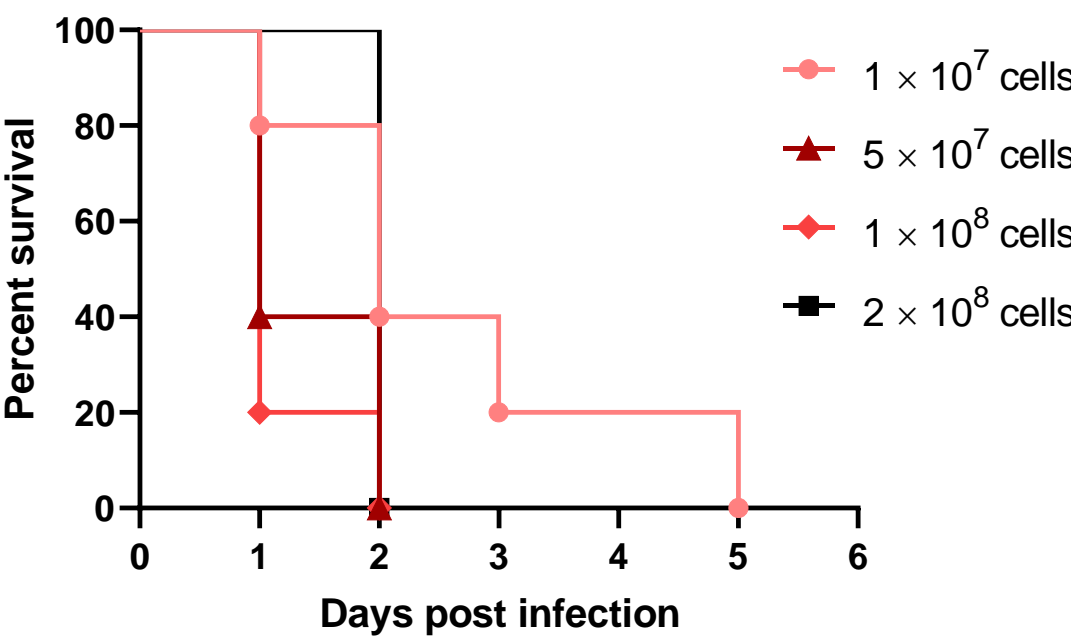

Supplement: FIG S8 [file mbio.02729-21-sf008.pdf]
