# Supplementary material for: Adenylyl Cyclase and Protein Kinase A Play Redundant and Distinct Roles in Growth, Differentiation, Antifungal Drug Resistance, and Pathogenicity of Candida auris
Source: mBio. 2021 Oct 19;12(5):e02729-21. doi: 10.1128/mBio.02729-21 (PMC8524339; doi:10.1128/mBio.02729-21)
Supplement: TABLE S1 [file mbio.02729-21-st001.docx]

**Table S1. *C. auris* strains used in this study**

| **Strain** | **Genotype** | **Parent** | **Reference** |
| --- | --- | --- | --- |
| B8441 | Wild-type |  | (1) |
| YSBA4 | *bcy1*Δ::*NAT* | B8441 | This study |
| YSBA6 | *bcy1*Δ::*NAT* | B8441 | This study |
| YSBA13 | *tpk1*Δ::*NAT* | B8441 | This study |
| YSBA14 | *tpk1*Δ::*NAT* | B8441 | This study |
| YSBA16 | *tpk2*Δ::*NAT* | B8441 | This study |
| YSBA17 | *tpk2*Δ::*NAT* | B8441 | This study |
| YSBA21 | *cyr1*Δ::*NAT* | B8441 | This study |
| YSBA23 | *cyr1*Δ::*NAT* | B8441 | This study |
| YSBA24 | *tpk2*Δ::*NAT tpk1*Δ::*HYG* | YSBA17 | This study |
| YSBA25 | *tpk2*Δ::*NAT tpk1*Δ::*HYG* | YSBA17 | This study |
| YSBA26 | *tpk2*Δ::*TPK2-HYG* | YSBA17 | This study |
| YSBA29 | *bcy1*Δ::*BCY1-HYG* | YSBA4 | This study |
| YSBA36 | *tpk1*Δ::*TPK1-HYG* | YSBA13 | This study |
| YSBA38 | *cyr1*Δ+*CYR1-HYG* | YSBA21 | This study |

**References**

1. **Munoz JF, Gade L, Chow NA, Loparev VN, Juieng P, Berkow EL, Farrer RA, Litvintseva AP, Cuomo CA**. 2018. Genomic insights into multidrug-resistance, mating and virulence in *Candida auris* and related emerging species. Nat Commun **9**:5346.
