# Supplementary material for: Adenylyl Cyclase and Protein Kinase A Play Redundant and Distinct Roles in Growth, Differentiation, Antifungal Drug Resistance, and Pathogenicity of Candida auris
Source: mBio. 2021 Oct 19;12(5):e02729-21. doi: 10.1128/mBio.02729-21 (PMC8524339; doi:10.1128/mBio.02729-21)
Supplement: TABLE S2 [file mbio.02729-21-st002.docx]

**Table S2. Primers used in this study**

| Name | Primer description | Sequence (5’ to 3’) |
| --- | --- | --- |
| B10738 | *BCY1* 5’-flanking region primer L1 | AGAGGCTTGAACCATTCC |
| B10739 | *BCY1* 5’-flanking region primer L2 | CAGATCCACTAGTTCTAGAACTCCTCTGTAGCGAACG |
| B10740 | *BCY1* 3’-flanking region primer R1 | TCCAGAATTTCACTCTTACCAGCACTTGATTTGTTGAG |
| B10741 | *BCY1* 3’-flanking region primer R1 | ACAGATGAAAGCCTCAACC |
| B10742 | *BCY1* 5’-screening primer SO | GGTTTTGCTTCTTCTGCG |
| B13115 | *BCY1* 3’-screening primer SO2 | TTGTATACTTGCAAGTTATC |
| B10743 | *BCY1* Southern blot probe primer PO | GGCTTCAAGATGAGGAAAGAG |
| B13116 | *BCY1* Internal screening primer LP | GGAGTTGGAGCAGTTGCAGA |
| B10807 | *TPK2* 5’-flanking region primer L1 | GGAAGAGATGACTGAAACCTC |
| B10808 | *TPK2* 5’-flanking region primer L2 | CAGATCCACTAGTTCTAGGAATGAGGGGTCAGTTTAGTC |
| B10809 | *TPK2* 5’-flanking region primer R1 | TCCAGAATTTCACTCTTACGCCAATGAAGACATACG |
| B10810 | *TPK2* 5’-flanking region primer R2 | CTGAACAAGACCTATTGATGCC |
| B10811 | *TPK2* 5’-screening primer SO | GCATTGTTGTTCTCTGGTCAG |
| B12469 | *TPK2* 3’-screening primer SO2 | TTTCATCCTCCTTCAAAATG |
| B10812 | *TPK2* Southern blot probe primer PO | TCTCACCAAATGGACACG |
| B12470 | *TPK2* Internal screening primer LP | ACTGACCCCTCATTCCCAAG |
| B12471 | *TPK2* Internal screening primer RP | CGTGGTAATGACTTCTGGCG |
| B11317 | *TPK1* 5’-flanking region primer L1 | ACAAATACTGATGAATCTGA |
| B10802 | *TPK1* 5’-flanking region primer L2 | CAGATCCACTAGTTCTAGCCAGTTGAGTCTCGTGATAGTC |
| B10803 | *TPK1* 5’-flanking region primer R1 | TCCAGAATTTCACTCTTAGGATAAGGAACTTGACTACGG |
| B11318 | *TPK1* 5’-flanking region primer R2 | TGCTTCCAATGGTCCTATC |
| B10806 | *TPK1* 5’-screening primer SO | GAGCAACATCATCAGCAGTAG |
| B12485 | *TPK1* 5’-screening primer SO2 | AAGACTCGCATGAACCTCAA |
| B10805 | *TPK1* Southern blot primer PO | ACCTCTGGGATTTTCGTAAC |
| B12484 | *TPK1* Internal screening primer LP | TTTGGCAGAGTTCACTTGGC |
| B13182 | *TPK1* Internal screening primer LP | CCAGAAGTGATGGGAGGTTC |
| B11321 | *CYR1* 5’-flanking region primer L1 | TCCAAACTATTGCCACCG |
| B11322 | *CYR1* 5’-flanking region primer L2 | CAGATCCACTAGTTCTAGCTTAGACTTGTCTTTTCTAA |
| B11323 | *CYR1* 5’-flanking region primer R1 | TCCAGAATTTCACTCTTAAAGGAATGGTGGAAACGG |
| B11324 | *CYR1* 5’-flanking region primer R2 | GCGTGACAATGTATCTTGC |
| B11326 | *CYR1* 5’-screening primer SO | GCTCAGTGACAACGGTTTACTAC |
| B12335 | *CYR1* 5’-screening primer SO2 | AAAGATAGCTCATTCAAGAA |
| B11325 | *CYR1* Southern blot primer | TTGTTCTGAGGCACCTTCC |
| B12336 | *CYR1* Internal screening primer LP | AACTACAGAGGGTTCCACGG |
| B12337 | *CYR1* Internal screening primer LP | CCGAAGAGAATTGGAAGGCG |
| B12460 | *TPK1* 5’-flanking region primer L2 (HYG) | TTTTCGGGGAAATGTGTGAGTCTCGTGATAGTCCAT |
| B12461 | *TPK1* 5’-flanking region primer R1 (HYG) | GATGAGGTAGTGCAAGTTAGCGCTCTTCCCAGATTTTTA |
| B11103 | pV1025 forward-extended primer | CTAGAACTAGTGGATCTGAA |
| B11104 | pV1025 reverse-extended primer | TAAGAGTGAAATTCTGGAAA |
| B11105 | NAT spilt primer 1 | CCATTGACTAAGGTTTTCCC |
| B11106 | NAT spilt primer 2 | TTCAGTAGCCAAACCCATC |
| B11107 | pV1025 Diagnostic screening primer 1 | TCAGTGGCAAATCCTAACC |
| B11108 | pV1025 Diagnostic screening primer 2 | AGAGAAAATACCCGTGACG |
| B12462 | pYM70 forward-extended primer | CACATTTCCCCGAAAAGTGC |
| B12463 | pYM70 reverse-extended primer | TAACTTGCACTACCTCATCG |
| B12464 | HYG spilt primer 1 | TGCTGATTTGTCTCAAACTT |
| B12465 | HYG split primer 2 | TACCATTATCAGTCAAAACA |
| B12486 | pYM70 Diagnostic screening primer 1 | TGCGGCACAATTGAATAGGG |
| B12487 | pYM70 Diagnostic screening primer 2 | CGGTGATGACGGTGAAAACC |
| B13059 | CLP for *BCY1* complementation | ACCGGTTTCACTCACACATCTTTTGT |
| B13060 | CRP for *BCY1* complementation | ACCGGTGAAGATGCTACTATCAATGG |
| B13114 | Screening primer for *BCY1* complementation | TGAGTATGTGAAAAAAAGCA |
| B11639 | *BCY1* sequencing primer 1 | TGTCCTCCACACTTCCTCAG |
| B11640 | *BCY1* sequencing primer 2 | TTGTTCAAAGGAGGGTTCGGCG |
| B11641 | *BCY1* sequencing primer 3 | TCAGGCTTCACAAGGTTGC |
| B13033 | CLP for *TPK1* complementation | ACTAGTTCGGCTATGATTCGGCCGAT |
| B13034 | CRP for *TPK1* complementation | ACTAGTAGAAGACTGGGAAGTGGAGC |
| B12794 | Screening primer for *TPK1* complementation | ACTAGTCGCAGTGAAGAGCGTCACCC |
| B12086 | *TPK1* sequencing primer 1 | ATTTTCTTCCAACCTCTTGA |
| B12087 | *TPK1* sequencing primer 2 | TATTCCTTGCCATAGAGTAT |
| B12088 | *TPK1* sequencing primer 3 | GTACTCATATTAATTCTTGT |
| B12306 | CLP for *TPK2* complementation | GTCGACTTGTGGTGACACTCATGACC |
| B12832 | CRP for *TPK2* complementation | GGTACCTACACCGAGTTGGCTGAGAACC |
| B12960 | Screening primer for *TPK2* complementation | TCAGAGAGTACGACTTGAAG |
| B12444 | *TPK2* sequencing primer 1 | AGTGTCTCCGGTAAGTAGCC |
| B12445 | *TPK2* sequencing primer 2 | TACCGAGCAGGCTGACAGTG |
| B12446 | *TPK2* sequencing primer 3 | TCCCATAACTGCCGGTACAG |
| B12918 | CLP for *CYR1* complementation | ACTAGTTGCTGTGCCTCGGCAGGATT |
| B13045 | CRP for *CYR1* complementation | GGTACCTTCAATTGCTAAACCTTTGA |
| B12950 | CLP2 for *CYR1* complementation | GCTTAGCCTTCGACAAATCAAGAACT |
| B12951 | CRP2 for *CYR1* complementation | GCTAAGCTTACTATGTTGCCTTCCGA |
| B13378 | Screening primer for *CYR1* complementation | GCGATGCCTTCAAGCTGTGT |
| B12920 | *CYR1* sequencing primer 1 | TGGGGACACTTGCTAGTACT |
| B12921 | *CYR1* sequencing primer 2 | AATGACGATTCTGTACTCAA |
| B12922 | *CYR1* sequencing primer 3 | CCCTCAGGCTACTGTGATTG |
| B12924 | *CYR1* sequencing primer 5 | GGAGGGCTCGCGAATTTGAA |
| B12925 | *CYR1* sequencing primer 6 | TGGTAACATTGGTGACATCC |
| B12926 | *CYR1* sequencing primer 7 | GTTGTCAGTGAGAATGGGAA |
| B11749 | *ACT1* qRT-PCR primer 1 | TTGCTCCTGAAGAACACCCT |
| B11750 | *ACT1* qRT-PCR primer 2 | GCAGGAACGTTGAAGGTCTC |
| B12520 | *CDR1* qRT-PCR primer 1 | GGTGACGTTGTGTACTCTGC |
| B12521 | *CDR1* qRT-PCR primer 2 | CTTCACCCCTGTTTTGAGGC |
| B12522 | *CDR2* qRT-PCR primer 1 | CGCAGGGCACTTCTTCTTTT |
| B12523 | *CDR2* qRT-PCR primer 2 | CAAAAGTGATGCCGGTGTCA |
| B12524 | *MDR1* qRT-PCR primer 1 | GCAGGCATTGAATCGGATGT |
| B12525 | *MDR1* qRT-PCR primer 2 | CCCTCCCAGTCCACAAGAAT |
| B12556 | *MDR2* qRT-PCR primer 1 | AAGGGAAGCTACGGGATGAG |
| B12557 | *MDR2* qRT-PCR primer 2 | AGATTCTGCCGTTCACTGGA |
| B12554 | *ERG11* qRT-PCR primer 1 | TGCCCATCGTCTACAACCTT |
| B12555 | *ERG11* qRT-PCR primer 2 | TCTCTCTGCACAGCTCGAAA |
| B12563 | *TAC1* qRT-PCR primer 1 | GTGCCGCAGAAAGAAAGTGA |
| B12564 | *TAC1* qRT-PCR primer 2 | TCTTGGAAGGCTGTCTCTCG |
| B12660 | *FKS1* qRT-PCR primer 1 | CGAAGAACACGGTCAGGACA |
| B12661 | *FKS1* qRT-PCR primer 2 | CCTCAGGGGTCAAGACGTTC |
| B12909 | *ERG6* qRT-PCR primer 1 | AGAGACCAAGAGTTCGCCAA |
| B12910 | *ERG6* qRT-PCR primer 2 | TTAGCAACGTCAGCAGCATC |
| B12911 | *ERG3* qRT-PCR primer 1 | CGGCCAGTACATGTCGAATG |
| B12912 | *ERG3* qRT-PCR primer 2 | CCAATCTGTCCCACAACGTG |
| B12994 | *HSP90* qRT-PCR primer 1 | TTCCGCTGGTGCCGATGTCT |
| B12995 | *HSP90* qRT-PCR primer 2 | GGACGTGGTCGGCAACCAAGAA |
| B13237 | *EFG1* qRT-PCR primer 1 | TCGTCTACTGCTCCCTCGAT |
| B13238 | *EFG1* qRT-PCR primer 2 | CTTGCCTGGAGTAGGAGCAC |
| B13235 | *UME6* qRT-PCR primer 1 | ATGAGACGAAACCCAGTTGC |
| B13236 | *UME6* qRT-PCR primer 2 | GCTTTTTGCGTCGAAGAGTC |
| B13289 | *CHS1* qRT-PCR primer 1 | GCCTGAAAGTATCCCGGAGT |
| B13290 | *CHS1* qRT-PCR primer 2 | CCAAATCCTAGTCGCATGCC |
| B13291 | *CHS2* qRT-PCR primer 1 | CGGCAGAACAGTTTACGACC |
| B13292 | *CHS2* qRT-PCR primer 2 | GGGCTTCTGTCTCACCTCTT |
| B13293 | *CHS3* qRT-PCR primer 1 | GGAGAGAGAAGATGGGGCTC |
| B13294 | *CHS3* qRT-PCR primer 2 | GTGGTATTGTGGCAAACGGT |
| B13295 | *CHS4* qRT-PCR primer 1 | GGGTGAAGTTGTCGAATCGG |
| B13296 | *CHS4* qRT-PCR primer 2 | GGCACAGATGGAGAGCATTG |
| B13297 | *CHS5* qRT-PCR primer 1 | GTGGGTAAACTCGATGCGTC |
| B13298 | *CHS5* qRT-PCR primer 2 | TAACAATCGAGCCGGCTTTG |
| B13299 | *CHS6* qRT-PCR primer 1 | TGCACACATACATTGGCGAG |
| B13300 | *CHS6* qRT-PCR primer 2 | CTGGATCGTCTGCACACATG |
| B13301 | *CHS7* qRT-PCR primer 1 | GGTATTGTGGGTGCCTTGTG |
| B13302 | *CHS7* qRT-PCR primer 2 | ATACCCACATCGACCGAGTC |
| B13303 | *CHS8* qRT-PCR primer 1 | TCGAGTACAATGGTCCCGAG |
| B13304 | *CHS8* qRT-PCR primer 2 | CCACGCTCATTCTGTTAGGC |
| B13305 | *CDA2* qRT-PCR primer 1 | CAGCTCCAGTGGTCGATTTG |
| B13306 | *CDA2* qRT-PCR primer 2 | TTGCACGAACTCTGTTGTCG |
| B13554 | *GPD1* qRT-PCR primer 1 | CCCACCAGTTCTTACCCAAA |
| B13555 | *GPD1* qRT-PCR primer 2 | TCAGGAGTCACCTCCAAACC |
| B13556 | *SOD1* qRT-PCR primer 1 | CATCCACCAGTTTGGTGACA |
| B13557 | *SOD1* qRT-PCR primer 2 | TGTCTGTTGTCGTCCTCTGG |
| B13558 | *CAT1* qRT-PCR primer 1 | TGTTCGAGCACGTTGGTAAG |
| B13559 | *CAT1* qRT-PCR primer 2 | CTTGGTGGAGAAGCCTCTTG |
| B13560 | *SRX1* qRT-PCR primer 1 | CCCGGTGCTAGACTACCAGA |
| B13561 | *SRX1* qRT-PCR primer 2 | GGCAACTCTCCTGGTGTGAT |
| B13562 | *GPX1* qRT-PCR primer 1 | GCCTCCAAATGTGGCTTTAC |
| B13563 | *GPX1* qRT-PCR primer 2 | GCCGAACTGGTTACAAGGAA |
| B13564 | *TSA1* qRT-PCR primer 1 | GACCGCTATCATCCAGAAGC |
| B13565 | *TSA1* qRT-PCR primer 2 | ACCCACTTGCCCTTGTACTG |
| B13566 | *TRX1* qRT-PCR primer 1 | GTTGACGAGGTTGGTGAGGT |
| B13567 | *TRX1* qRT-PCR primer 2 | GTTGGCACCAATCACCTTCT |
| B13576 | *GPD2* qRT-PCR primer 1 | TCCGTGGTGAAGGTAAGGAC |
| B13577 | *GPD2* qRT-PCR primer 2 | CCAGCAATAGAGGCACCAAT |
| B13578 | *GPP1* qRT-PCR primer 1 | CACGTGAACCCAGAGGAAAT |
| B13579 | *GPP1* qRT-PCR primer 2 | TTCGGTCACCTGCTTTTCTT |
| B13580 | *ENA1* qRT-PCR primer 1 | TCTGCGCTATGACTGGTGAC |
| B13581 | *ENA1* qRT-PCR primer 2 | GGACGCATCCTTAGCAACAT |
| B13582 | *TTR1* qRT-PCR primer 1 | TCAAACAGAGCTCGGGAAAC |
| B13583 | *TTR1* qRT-PCR primer 2 | GGCCTCTTCCTTTCCTTTTG |
| B13584 | *TRR1* qRT-PCR primer 1 | TGTGGACTGAGTGGAACGAA |
| B13585 | *TRR1* qRT-PCR primer 2 | CTTGTCCTCACCTGGGATGT |
| B14115 | *HSF1* qRT-PCR primer 1 | CAAAACCAGCATTCGTGATG |
| B14116 | *HSF1* qRT-PCR primer 2 | TGGAATGTTAAGCCGTCCTC |
| B14117 | *HSP12* qRT-PCR primer 1 | CTTCTAGCCAGCCATCCTTG |
| B14118 | *HSP12* qRT-PCR primer 2 | CGCTGACGACTTGAGAAACA |
| B14119 | *HSP70* qRT-PCR primer 1 | TGGGAACCACGTATTCCTGT |
| B14120 | *HSP70* qRT-PCR primer 2 | AGGCAACGTATGAGGGTGTC |
| B14121 | *HSP78* qRT-PCR primer 1 | GAACCGTTTGGACGAAGTGT |
| B14122 | *HSP78* qRT-PCR primer 2 | TCGCCAATCTCTCTCAACCT |
| B14123 | *HSP104* qRT-PCR primer 1 | GGATGGTGCCTTTGAAAGAA |
| B14124 | *HSP104* qRT-PCR primer 2 | AGGCTGCAAACCTCTCAAAA |
| B14578 | *FLO8* qRT-PCR primer 1 | ACAGAGCCAGAACGGAGCTA |
| B14579 | *FLO8* qRT-PCR primer 2 | GCTTCTCCAAAAGCATGAGC |
| B14580 | *WOR1* qRT-PCR primer 1 | TTGCCGCCTTATCAGAACTT |
| B14581 | *WOR1* qRT-PCR primer 2 | TAGTGCTCGTCCTGCTGAAA |
| B14920 | *PRX1* qRT-PCR primer 1 | GACGAGATCACTGGCTCACA |
| B14921 | *PRX1* qRT-PCR primer 2 | GTGGCGTCCTGGTAGTCAAT |
